# Characterization of cardiac involvement in children with *LMNA*-related muscular dystrophy

**DOI:** 10.3389/fcell.2023.1142937

**Published:** 2023-03-10

**Authors:** Sergi Cesar, Oscar Campuzano, Jose Cruzalegui, Victori Fiol, Isaac Moll, Estefania Martínez-Barrios, Irene Zschaeck, Daniel Natera-de Benito, Carlos Ortez, Laura Carrera, Jessica Expósito, Rubén Berrueco, Carles Bautista-Rodriguez, Ivana Dabaj, Marta Gómez García-de-la-Banda, Susana Quijano-Roy, Josep Brugada, Andrés Nascimento, Georgia Sarquella-Brugada

**Affiliations:** ^1^ Pediatric Arrhythmias, Inherited Cardiac Diseases and Sudden Death Unit, Hospital Sant Joan de Déu, Universitat de Barcelona, Barcelona, Spain; ^2^ Arrítmies Pediàtriques, Cardiologia Genètica i Mort sobtada, Malalties Cardiovasculars en el Desenvolupament, Institut de Recerca Sant Joan de Déu, Esplugues de Llobregat, Barcelona, Spain; ^3^ European Reference Network for Rare, Low Prevalence and Complex Diseases of the Heart (ERN GUARD-Heart), Amsterdam, Netherlands; ^4^ Medical Science Department, School of Medicine, Universitat de Girona, Girona, Spain; ^5^ Cardiovascular Genetics Center, University of Girona-IDIBGI, Girona, Spain; ^6^ Centro de Investigación Biomédica en Red, Enfermedades Cardiovasculares (CIBERCV), Madrid, Spain; ^7^ Neuromuscular Unit, Department of Neurology, Hospital Sant Joan de Déu, Barcelona, Spain; ^8^ Investigación Aplicada en Enfermedades Neuromusculares, Neurociències, Institut de Recerca Sant Joan de Déu, Esplugues de Llobregat, Spain; ^9^ Servicio de Hematología Pediátrica, Hospital Sant Joan de Déu Barcelona, Institut de Recerca Pediàtrica, Hospital Sant Joan de Déu de Barcelona (IRP-HSJD), Universitat de Barcelona, Barcelona, Spain; ^10^ Paediatric Cardiology Services, Royal Brompton Hospital, Guy’s and St Thomas NHS Foundation Trust, London, United Kingdom; ^11^ National Heart and Lung Institute, Imperial College London, London, United Kingdom; ^12^ Neuromuscular Unit, Pediatric Neurology and ICU Department, Raymond Poincaré Hospital (UVSQ), AP-HP Université Paris-Saclay, Garches, France; ^13^ Arrhythmia Section, Cardiology Service, Hospital Clínic, Barcelona, Spain; ^14^ Instituto Nacional de Investigación Biomédica de Enfermedades Raras (CIBERER), Instituto de Salud Carlos III, Madrid, España

**Keywords:** laminopathies, sudden cardiac death, cardiomyopathy, A/C lamins, *LMNA*-related diseases, LMNA-related cardiomyopathy, long-term implantable loop recorder

## Abstract

**Introduction:** LMNA-related muscular dystrophy is a rare entity that produce “laminopathies” such as Emery–Dreifuss muscular dystrophy (EDMD), limb–girdle muscular dystrophy type 1B (LGMD1B), and LMNA-related congenital muscular dystrophy (L-CMD). Heart failure, malignant arrhythmias, and sudden death may occur. No consensus exists on cardiovascular management in pediatric laminopathies. The aim was to perform an exhaustive cardiologic follow-up in pediatric patients diagnosed with LMNA-related muscular dystrophy.

**Methods:** Baseline cardiac work-up consisted of clinical assessment, transthoracic Doppler echocardiography, 12-lead electrocardiogram, electrophysiological study, and implantation of a long-term implantable cardiac loop recorder (ILR).

**Results:** We enrolled twenty-eight pediatric patients diagnosed with EDMD (13 patients), L-CMD (11 patients), LGMD1B (2 patients), and LMNA-related mild weakness (2 patients). Follow-up showed dilated cardiomyopathy (DCM) in six patients and malignant arrhythmias in five (four concomitant with DCM) detected by the ILR that required implantable cardioverter defibrillator (ICD) implantation. Malignant arrhythmias were detected in 20% of our cohort and early-onset EDMD showed worse cardiac prognosis.

**Discussion:** Patients diagnosed with early-onset EDMD are at higher risk of DCM, while potentially life-threatening arrhythmias without DCM appear earlier in L-CMD patients. Early onset neurologic symptoms could be related with worse cardiac prognosis. Specific clinical guidelines for children are needed to prevent sudden death.

## Introduction

*LMNA*-related muscular dystrophy is a very rare (0.5 per 100,000) disease caused by pathogenic alterations in the *LMNA* gene. The disorder is characterized by cervical–axial weakness, scapuloperoneal weakness, joint contractures, thoracic lordosis, a dystrophic muscle biopsy, and mildly elevated creatine kinase levels (Quijano-roy et al., 2008). Children with early-onset *LMNA*-related muscular dystrophy may show decreased fetal movement and early lack of motor development since the first months of life, or later develop a loss of head and trunk control and ability to walk or sit, followed by progressive loss of axial and limb motor function. As these patients age, there is an increased risk for respiratory insufficiency, appears joint and spinal deformities and cardiac involvement. Practically all patients exhibit heart disease in long follow up studies (Dubowitz, 1999; Quijano-roy et al., 2008).

*LMNA* encodes the nuclear envelope proteins lamins A and C, intermediate filaments that are required during development and cell differentiation (Bonne et al., 2003). Lamins facilitate signal transduction between the cytoskeleton and the nucleus (Aebi et al., 1986; Worman and Bonne, 2007), provide genome stability and modulation of chromatin organization and gene expression (Lammerding et al., 2004; Dechat et al., 2007; Andrés and González, 2009; Dauer and Worman, 2009; Gonzalez-Suarez et al., 2009; Hutchison, 2011). Lamins consist of a globular N-terminal head domain, a central coiled-coil rod domain implicated in protein dimerization, and a C-terminal tail domain that includes an immunoglobulin-like domain where various posttranslational modifications occur (Burke and Stewart, 2012; Ho and Lammerding, 2012). *LMNA* was first identified in 1986 in humans, but it was not until 1999 that a pathogenic rare variant in the *LMNA* gene was linked to Emery–Dreifuss muscular dystrophy (EDMD) (Bonne et al., 1999). To date, over 600 disease-causing rare *LMNA* variants are characterized (Worman and Bonne, 2007; Dittmer and Misteli, 2011), and these “laminopathies” are associated with heterogeneous clinical phenotypes, including neuromuscular, cardiac, and metabolic disorders (Bertrand et al., 2011; Worman, 2012; Maggi et al., 2014). However, there is no clear correlation between genotype and phenotype, including within the same muscle, suggesting the presence of genetic modifiers and representing an example of allelic heterogeneity (Granger et al., 2011).

Muscle laminopathies may associate cardiac disease at any age and range from congenital muscular dystrophy (*LMNA*-related congenital muscular dystrophy, or L-CMD) to late-onset manifestations (limb–girdle muscular dystrophy 1B, or LGMD1B; and autosomal-dominant EDMD). L-CMD is the most severe and early phenotype, and typically presents in the first 2 years of life, either by an arrest of motor milestones before sitting or walking are acquired, or as a later presentation with a characteristic loss of head support, while sitting and or walking are still maintained (dropped head syndrome). It is a very progressive and severe disease, and shares with EDMD a recognizable scapulo-humero-peroneal pattern of muscle weakness and atrophy (predominantly proximal in upper limbs and distal in lower limbs). The heart may be involved in all three entities, and the manifestation of heart disease may precede muscle weakness or be isolated. Globally, early-onset phenotypes before 5 years of age, and specially before 2 years of age, are related with worse motor and cardiac prognosis, although heart involvement is rarely observed initially (Granger et al., 2011; Carboni et al., 2013; Maggi et al., 2014; Ben Yaou et al., 2021). Dilated cardiomyopathy (DCM) with conduction disease and sudden cardiac death (SCD) can occur in *LMNA*-related muscular dystrophies, in children and adults (Finsterer et al., 2006; Finsterer et al., 2010; Lu et al., 2011; Groh, 2012; Quarta et al., 2012; Cattin et al., 2013; Hasselberg et al., 2014; Alastalo et al., 2015; Dobrzynska et al., 2016; Finsterer and St, 2016; Heller et al., 2017; Muscogiuri, 2017; Wang et al., 2017; Groh et al., 2022). These patients have a high incidence of malignant arrhythmias at early ages, worsening prognosis and posing a clinical challenge for cardiologists, neurologists, and genetic counselors (Meune et al., 2006; Groh, 2012; Rijsingen et al., 2012; Carboni et al., 2013; Rajdev and Groh, 2015; Kumar et al., 2016; Feingold et al., 2017; Peretto et al., 2019; Ben Yaou et al., 2021). Despite the risk prediction scores for life-threatening ventricular tachyarrhythmias (VT) have been defined in adults with laminopathies (Wahbi et al., 2019; Marchel et al., 2021a; Groh et al., 2022), there are no published guidelines for risk prediction scores in pediatric laminopathies to prevent early life-threatening VT. Given the rarity of the disease in the pediatric population, phenotype–genotype correlations are difficult to be established, and data on age of onset and course of the cardiac disease or the risk of malignant arrhythmias and SCD is scarce (Ben Yaou et al., 2021).

To bridge this knowledge gap, we performed a mid-to-long-term cardiovascular follow-up study focused on cardiomyopathies and arrhythmia during childhood and genotype–phenotype correlation in several families with *LMNA*-related muscular dystrophy.

## Material and methods

### Study design

We enrolled patients <18 years of age from the international reference center in neuromuscular diseases at our institution that were diagnosed with *LMNA*-related muscular dystrophy (carrying a rare pathogenic or likely pathogenic variant in the *LMNA* gene with L-CMD, EDMD, LGMD1B, and *LMNA*-related atypical phenotype with mild weakness) between 2014 and 2020. The study was motivated and promoted by the national patient association. Due to the severity of pediatric laminopathies, we analyzed in an independent category all the patients with early-onset neuromuscular phenotypes (before 2 years of age) regardless of the final diagnosed clinical phenotype (L-CMD and early-onset EDMD). At enrollment, we collected retrospective clinical data. Participants were prospectively followed from enrollment to the last follow-up evaluation according to a specific schedule. Written informed consent to participate was provided by the participants’ legal guardians. Non-LMNA-related neuromuscular dystrophies were excluded from this study.

### Clinical work-up and follow-up schedule

Baseline cardiac work-up consisted of a clinical evaluation, DNA samples from the patient and first-degree relatives, transthoracic Doppler echocardiography (Philips, IE33, software Intellispace Cardiovascular for standard measurements and QLAB for offline strain analysis), a 12-lead electrocardiogram (ECG), electrophysiological study (EPS), and implantation of a long-term cardiac implantable loop recorder (ILR; Medtronic Reveal LINQ) with a home monitoring system. The definition of DCM and reference values of echocardiographic measurements were adopted from the most recent pediatric guidelines and normal values published for children (Lang et al., 2005; Pettersen et al., 2008; Lee et al., 2014; Lee et al., 2017; Lipshultz et al., 2019).

EPS was performed in all patients at the time of inclusion to exclude underlying arrhythmogenic conditions and arrhythmia inducibility and to describe cardiac conduction system characteristics. The procedure was performed under mild sedation *via* the brachial or femoral vein (depending on joint retractions and grade of hyperlordosis). Baseline neuromuscular evaluation consisted of a detailed and standardized neurologic examination with diagnostic tests based on clinical indications. Retrospective clinical data from the referral center were collected. During follow-up, cardiologic and neurologic data were collected at least once a year; arrhythmia events were reviewed daily by long-term ILR software using a home monitoring system. Medical therapy and device implantation (pacemaker [PM] and implantable cardioverter defibrillator [ICD]) were indicated according to current clinical evidence.

For the echocardiogram analysis, a single observer obtained the following measurements at enrollment and during the follow-up: M-mode-derived left ventricular end-diastolic and end-systolic dimensions, left ventricular ejection fraction (LVEF; %) measured by the Simpson method, tricuspid annular plane systolic excursion (TAPSE; mm), mitral annular plane systolic excursion (MAPSE; mm), tissue doppler values from lateral and septal mitral annulus (cm/s), and spectral doppler E and A waves from inflow mitral and tricuspid filling pattern (cm/s). Global longitudinal strain (GLS [%]) was obtained from four-chamber view, and reference values (mean and p5) were obtained from published pediatric data (Koopman et al., 2019).

### Cardiac events

Major cardiac events included cardiac death, heart transplant, and malignant arrhythmias, which were defined as sustained VT, ventricular fibrillation (VF), asystole, complete atrioventricular block (AVB), cardiac arrest from VT/VF (witnessed SCD occurring within 1 h of acute symptoms), or appropriate treatment (antitachycardia pacing or shock) by ICD. Minor cardiac events included worsening of heart failure, reaching DCM criteria, conduction system abnormalities (except complete AVB), supraventricular tachycardia, or any structural or functional echocardiographic abnormality according to updated definitions and references. The timing of each event was reported as age, years from clinical onset, and months from study enrollment.

### Statistical analysis

Data were anonymized and stored in local institutions. We used StatCrunch (Pearson Education Inc.) for the statistical analysis. Categorical variables were expressed as numbers and percentages, and continuous variables were expressed as median and interquartile range (IQR). When appropriate, echocardiographic data were analyzed comparing enrollment and last control measurements. Because the data were not normally distributed, a Mann–Whitney *U* non-parametric test was used, as appropriate, for analysis of quantitative variables between two groups. A *p*-value of <0.05 was considered statistically significant. Graphical presentations of the major and minor events that occurred during follow-up were generated with GraphPad Software (Prism, version 9.1.1).

## Results

Twenty-eight individuals (median age of 8.5 years at enrollment; IQR of 4–12.5 years) from 27 families were enrolled. Table 1 summarizes the main clinical and genetic features of our cohort (Bonne et al., 2000a; Quijano-roy et al., 2008; Chemla et al., 2010; Komaki et al., 2011; Pasqualin et al., 2014; Heller et al., 2017; Fan et al., 2020). The median age at last follow-up was 13 years (IQR of 8–17 years). All participants met clinical criteria and had confirmed *LMNA*-related neuromuscular disease. 13 (46.43%) had EDMD, 11 had L-CMD (39.28%), 2 (7.14%) had LGMD1B, and 2 (7.14%) had an *LMNA*-related atypical phenotype with mild weakness (Supplementary Figure S1). All had a pediatric onset of skeletal muscle symptoms, with most of them presenting before 2 years of age (23 of the 28 cases, 82%).

**TABLE 1:** Clinical and genetic data.

Patient	Gender	Neuromuscular phenotype	Early onset	DCM	ICD/PM	Drugs	Death	*LMNA de novo*	*LMNA* c	*LMNA* p	Previously reported
1	M	L-CMD	Y	N	Y (ICD)	Carvedilol	N	Y	c.745C>T	p.Arg249Trp	Quijano, 2008/Komaki, 2011/Pasqualin, 2014/Chemla, 2010/Heller, 2017/Fan, 2021
						ASA					
2	M	L-CMD	Y	N	Y (PM)	N	N	Y	c.745C>T	p.Arg249Trp	Quijano, 2008/Komaki, 2011/Pasqualin, 2014/Chemla, 2010/Heller, 2017/Fan, 2021
3	F	EDMD	Y	Y	Y ICD)	Carvedilol Captopril Amiodarone	Y	Y	c.116A>G	p.Asn39Ser	Pasqualin, 2014/Fan, 2021
						Furosemide					
						Spironolactone					
						ASA					
						LMWH					
4	F	L-CMD	Y	N	N	N	N	Y	c.91-93delGAG	p.Glu31del	Fan, 2021
5	M	EDMD	Y	Y	Y ICD)	Sotalol	N	Y	c.1358G>C	p.Arg453Pro	N
						Flecainide					
						ASA					
6	M	L-CMD	Y	N	N	N	N	Y	c.745C>T	p.Arg249Trp	Quijano, 2008/Komaki, 2011/Pasqualin, 2014/Chemla, 2010/Heller, 2017/Fan, 2021
7	M	EDMD	Y	N	N	N	N	Y	c.116A>G	p.Asn39Ser	Pasqualin, 2014/Fan, 2021
8	M	EDMD	N	N	N	N	N	Y	c.746G>A	p.Arg249Gln	Bonne, 2000/Komaki, 2011/Fan, 2021
9	M	EDMD	Y	Y	N	Carvedilol	N	Y	c.91delG	p.Glu31ArgfsTer65	N
10	F	L-CMD	Y	N	N	N	N	Y	c.89A>C	P.Gln30Pro	N
11	F	L-CMD	Y	Y	N	N	N	N	c.1487_1488+9del	-	N
12	M	EDMD	Y	N	N	N	N	Y	c.1616C>T	p.Ala539Val	N
13	M	EDMD	Y	Y	Y ICD)	Carvedilol	N	Y	c.112C>T	p.Leu38Phe	N
						Flecainide					
						ASA					
14	F	EDMD	Y	N	N	N	N	Y	c.745C>T	p.Arg249Trp	Quijano, 2008/Komaki, 2011/Pasqualin, 2014/Chemla, 2010/Heller, 2017/Fan, 2021
15	M	EDMD	N	N	N	N	N	Y	c.812T>G	p.Leu271Arg	N
16	M	Mild Weakness	N	N	N	N	N	Y	c.879_881delCAG	p.Gln294del	N
17	M	EDMD	Y	Y	Y ICD)	Carvedilol	N	Y	c.1364G>C	p.Arg455Pro	N
18	M	Mild Weakness	N	N	N	N	N	Y	c.745C>T	p.Arg249Trp	Quijano, 2008/Komaki, 2011/Pasqualin, 2014/Chemla, 2010/Heller, 2017/Fan, 2021
19	F	LGMD1B	Y	N	N	N	N	Y	c.810 + 1G>C	-	N
20	F	EDMD	Y	N	N	N	N	N	c.108G>T	p.Gln36His	N
21	M	L-CMD	Y	N	N	N	N	Y	c.104T>A	p.Leu35Gln	N
22	F	EDMD	N	N	N	N	N	Y	c.1357C>T	p.Arg453Trp	Fan, 2021/Bonne, 2000
23	M	LGMD1B	Y	N	N	N	N	Y	c.1357C>T	p.Arg453Trp	Fan, 2021/Bonne, 2000
24	F	EDMD	Y	N	N	Flecainide	Y	Y	c.91G>A	p.Glu31Lys	Fan, 2021
25	F	L-CMD	Y	N	N	N	N	Y (twin)	c.117T>G	p.Asn39Lys	Fan, 2021
26	F	L-CMD	Y	N	N	N	N	Y (twin)	c.117T>G	p.Asn39Lys	Fan, 2021
27	M	L-CMD	Y	N	N	N	N	Y	c.94_96delAAG	p.Lys32del	Fan, 2021
28	F	L-CMD	Y	N	N	N	N	Y	c.745C>T	p.Arg249Trp	Quijano, 2008/Komaki, 2011/Pasqualin, 2014/Chemla, 2010/Heller, 2017/Fan, 2021

List of *LMNA*-related muscular dystrophy patients, major cardiac events, and rare *LMNA* variants. Abbreviations: L-CMD, *LMNA*-related congenital muscular dystrophy; EDMD, Emery–Dreifuss muscular dystrophy; LGMD1B, Limb–girdle muscular dystrophy 1B; DCM, dilated cardiomyopathy; ICD, implantable cardioverter defibrillator; PM, pacemaker; LMWH, low-molecular-weight heparin; ASA, acetylsalicylic acid

**FIGURE 1:**
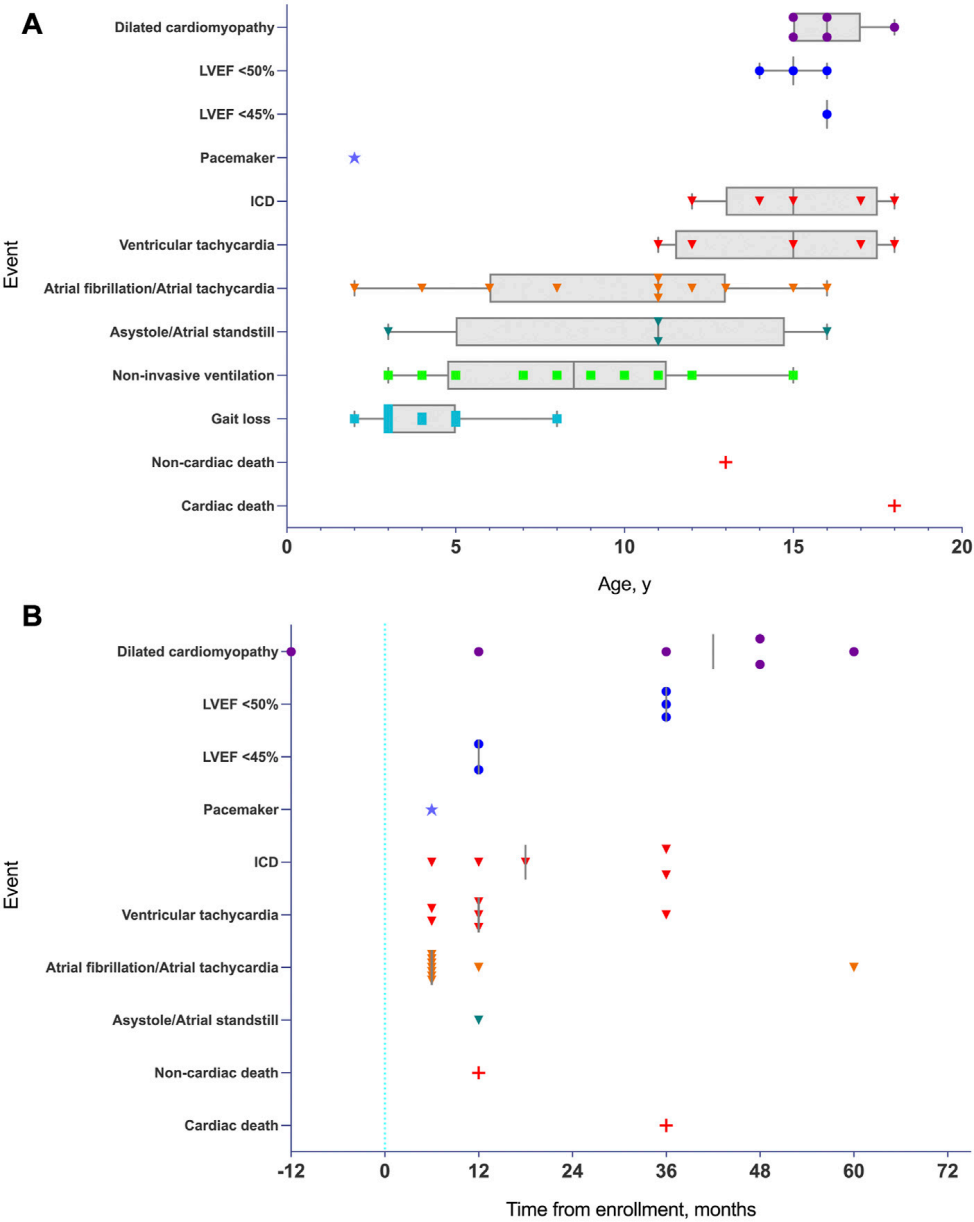
**(A)**: Timeline of major and minor events in pediatric patients with lamin-related muscular dystrophy defined on the basis of age. Atrial arrhythmias, asystole/atrial standstill, and malignant arrhythmias preceded structural and cardiac functional abnormalities (DCM criteria or depressed LVEF). Median time values are represented by vertical grey line when required. DCM and LVEF are represented by a dot. Pacemaker are represented by a star. Arrhythmias and ICD are represented by an inverted triangle. Gait loss and NIMV are represented by a square, and death are represented by a cross. When required, minimum to maximum range age is represented in grey box and whiskers. Abbreviations: LVEF, left ventricular ejection fraction. **(B)**: Timeline of major and minor events in pediatric patients with lamin-related muscular dystrophy defined on the basis of enrollment (time 0 is the time of enrollment represented by a vertical blue dotted line). Median time values are represented by a vertical grey line. DCM and LVEF are represented by a dot, pacemaker is represented by a star, arrhythmias and ICD are represented by an inverted triangle and death is represented by a cross. Abbreviations: LVEF, left ventricular ejection fraction.

The median follow-up from study enrollment to last clinical evaluation was 4 years (IQR of 3–5 years) (Supplementary Figure S2). All living participants (except eight patients) had a comprehensive cardiac follow-up every 6 months that included echocardiography and remote cardiac rhythm monitoring. In the other eight patients, only clinical data and remote cardiac rhythm monitoring were available. Events by age and events by enrollment date are represented graphically in Figure 1 (Figures 1A, B).

A total of 57.1% of participants were male. Two of the cases enrolled were female monozygotic twins. Families were originally from Spain (Bonne et al., 1999), the United Kingdom (Bonne et al., 2003), the United States (Bonne et al., 2003), Australia (Dubowitz, 1999), Canada (Quijano-roy et al., 2008), France (Quijano-roy et al., 2008), Greece (Quijano-roy et al., 2008), Russia (Quijano-roy et al., 2008), and Venezuela (Quijano-roy et al., 2008). Eleven patients (39.29%) had L-CMD, 13 (46.43%) had EDMD, 2 (7.14%) had LGMD1B, and 2 (7.14%) had an *LMNA*-related atypical phenotype with mild weakness. Early-onset skeletal muscle impairment before 2 years of age was detected in 23 of the 28 cases (82%). Table 1 summarizes the main clinical and genetic features of our cohort (Bonne et al., 2000a; Quijano-roy et al., 2008; Chemla et al., 2010; Komaki et al., 2011; Pasqualin et al., 2014; Heller et al., 2017; Fan et al., 2020). Eleven patients (39.2%; seven males) required non-invasive mechanical ventilation (NIMV) either at inclusion or during follow-up. Patients requiring NIMV had early-onset EDMD and L-CMD phenotypes. For those with gait loss, the median age for gait loss was 6 years (IQR of 5–8 years). Supplementary Table S1 compares our cohort with previous publications not focused on pediatric patients.

### Major and minor cardiac events during follow-up

#### Cardiac function and structural heart disease

Table 2 and Supplementary Table S2 summarize major and minor cardiac events and statistical analyses of the echocardiographic data. During follow-up, rapid progression to DCM was seen in six cases (21.4%). (patient 3 [died], 5, 9, 11, 13, and 17), all showing early neuromuscular impairment before 2 years of age; five were diagnosed with early-onset EDMD, and one was diagnosed with L-CMD (patient 11).

**TABLE 2:** Demographic data and overall follow-up.

Patient	Neuromuscular phenotypes	Early onset	Follow-up (time, years)	Gender	Age (y) at enroll/end follow-up	NIMV age (y)	Gait loss age (y)	LVEF simpson (%)		4-Chamber GLS (%)		ECG features during follow-up	Implantable loop recorder		DCM at end of follow-up	ICD/PM	Death	Drugs	LMNA c	LMNA p
													Atrial events	Ventricular events						
								Enroll	Final	Enroll	Final									
1	L-CMD	Y	4	M	11/15	12	8	63	54	−21	−20.6	Wide QRS	AT,AF	NSVT	N	Y (ICD)	N	Carvedilol	c.745C>T	p.Arg249Trp
												1st degree AVB	Atrial standstill					ASA		
2	L-CMD	Y	6	M	2/8	N/A	3	59	57	−22	−19.5	High QRS voltage (V2-V4)	AT	-	N	Y (PM)	N	N	c.745C>T	p.Arg249Trp
3	EDMD	Y	2	F	16/18	15	3	50	26	−9	−7	1st degree AVB	AT,AF	SVT, VF	Y	Y ICD)	Y	Carvedilol	c.116A>G	p.Asn39Ser
												Wide QRS	Atrial standstill					Captopril		
												QS complex (V1)						Amiodarone		
												Poor R progression						Spironolactone		
												Repolarization abnormalities						ASA		
																		LMWH		
4	L-CMD	Y	3	F	9/12	5	3	54.7	69	−20.5	−17.6	N/A	AT	-	N	N	N	N	c.91-93delGAG	p.Glu31del
5	EDMD	Y	5	M	11/16	4	5	65	50	−21	−16.3	Wide QRS	AT,AF, Atrial standstill	NSVT	Y	Y ICD)	N	Sotalol	c.1358G>C	p.Arg453Pro
												IRBBB						Flecainide		
												1st degree AVB						ASA		
6	L-CMD	Y	5	M	9/14	9	5	51	N/A	−17.4	N/A	N/A	AT	-	N	N	N	N	c.745C>T	p.Arg249Trp
7	EDMD	Y	5	M	3/8	3	N/A	69	N/A	−21.5	N/A	N/A	AT	-	N	N	N	N	c.116A>G	p.Asn39Ser
8	EDMD	N	5	F	8/13	N/A	N/A	63	50	−19	−18.8	Global Low voltage	-	-	N	N	N	N	c.746G>A	p.Arg249Gln
9	EDMD	Y	5	M	7/12	N/A	N/A	55	N/A	−20	N/A	N/A	AT	-	Y	N	N	Carvedilol	c.91delG	p.Glu31ArgfsTer65
10	L-CMD	Y	4	F	2/6	N/A	N/A	60.5	55	−26.4	−25	High QRS voltage (V2-V4)	-	-	N	N	N	N	c.89A>C	P.Gln30Pro
												Q waves in III, V5-V6								
												Short PR segment								
11	L-CMD	Y	4	F	3/8	N/A	4	54	55	−22	−18	Normal	-	-	Y	N	N	N	c.1487_1488+9del	NA
12	EDMD	Y	3	M	15/18	N/A	N/A	59	61	−23.9	−17	Global low voltage	-	-	N	N	N	N	c.1616C>T	p.Ala539Val
												Short PR segment								
												Sinus bradycardia								
												Repolarization abnormalities								
13	EDMD	Y	4	M	15/19	10	4	58	52	−17.3	−13.7	Global low voltage	AT	NSVT	Y	Y ICD)	N	Carvedilol	c.112C>T	p.Leu38Phe
												IRBBB						Flecainide		
												Deep S wave in V3						ASA		
14	EDMD	Y	5	F	8/13	7	3	64	51.2	−23	−20.8	High QRS voltage (V4)	-	-	N	N	N	N	c.745C>T	p.Arg249Trp
15	EDMD	N	5	M	13/18	N/A	N/A	61	56.8	−19	−14.8	Global low voltage	-	-	N	N	N	N	c.812T>G	p.Leu271Arg
16	Mild Weakness	N	5	M	10/15	N/A	N/A	65	65	−21	−16.6	Normal	-	-	N	N	N	N	c.879_881delCAG	p.Gln294del
17	EDMD	Y	4	M	15/19	11	N/A	40	44	−13.6	−21	QS complex (V2)	-	NSVT	Y	Y ICD)	N	Carvedilol	c.1364G>C	p.Arg455Pro
												Poor R wave progression								
												Repolarization abnormalities								
18	Mild Weakness	N	3	M	18/21	N/A	N/A	68	N/A	−20	N/A	N/A	-	-	N	N	N	N	c.745C>T	p.Arg249Trp
19	LGMD1B	Y	5	F	11/16	N/A	N/A	61	N/A	23.8	N/A	N/A	-	-	N	N	N	N	c.810 + 1G>C	N/A
20	EDMD	Y	5	F	3/8	N/A	3	64	N/A	−19	N/A	N/A	AT,AF	NSVT	N	N	N	N	c.108G>T	p.Gln36His
21	L-CMD	Y	5	M	3/8	N/A	3	63	N/A	−20	N/A	Normal	-	-	N	N	N	N	c.104T>A	p.Leu35Gln
22	EDMD	N	4	F	17/21	N/A	N/A	58.5	56.9	−19	−19.2	Global low voltage	-	-	N	N	N	N	c.1357C>T	p.Arg453Trp
23	LGMD1B	Y	3	M	5/8	N/A	3	63	60	−22	−21.7	Normal	-	-	N	N	N	N	c.1357C>T	p.Arg453Trp
24	EDMD	Y	1	F	12/13	8	N/A	57	58	−19.5	−20	Global low voltage	AT	-	N	N	Y	Flecainide	c.91G>A	p.Glu31Lys
												Repolarization abnormalities								
25	L-CMD	Y	2	F	5/7	N/A	N/A	54.5	56.7	−22.6	−23.1	Global low voltage	-	-	N	N	N	N	c.117T>G	p.Asn39Lys
												Poor R progression								
26	L-CMD	Y	2	F	5/7	N/A	N/A	61.1	59.5	−22.6	−28.8	Global low voltage	-	-	N	N	N	N	c.117T>G	p.Asn39Lys
												Poor R progression								
27	L-CMD	Y	1	M	4/5	N/A	N/A	57.2	66.9	−26.5	N/A	Poor R progression	-	-	N	N	N	N	c.94_96delAAG	p.Lys32del
28	L-CMD	Y	1	M	4/5	N/A	N/A	68	N/A	−23.5	N/A	Global low voltage	-	-	N	N	N	N	c.745C>T	p.Arg249Trp

List of *LMNA*-related muscular dystrophy patients, major and minor cardiac events, and rare *LMNA* mutations. Abbreviations: L-CMD, *LMNA*-related congenital muscular dystrophy; EDMD, Emery–Dreifuss muscular dystrophy; LGMD1B, Limb–girdle muscular dystrophy 1B; M, male; F, female; DCM, dilated cardiomyopathy; ICD, implantable cardioverter defibrillator; PM, pacemaker; AT, atrial tachycardia; AF, atrial fibrillation; NSVT, non-sustained ventricular tachycardia; SVT, sustained ventricular tachycardia; VF, ventricular fibrillation; GLS, global longitudinal strain; N/A, not available; AVB, atrioventricular block; IRBBB, incomplete right bundle branch block

Two of these patients showed LVEF values of less than 45% at last follow-up, and four showed DCM requiring an ICD. No right ventricle (RV) involvement was detected in our cohort, but advanced RV myocardial strain analysis could not be adequately performed because of suboptimal image quality from the RV related to chest wall deformities in the majority of patients.

**FIGURE 2:**
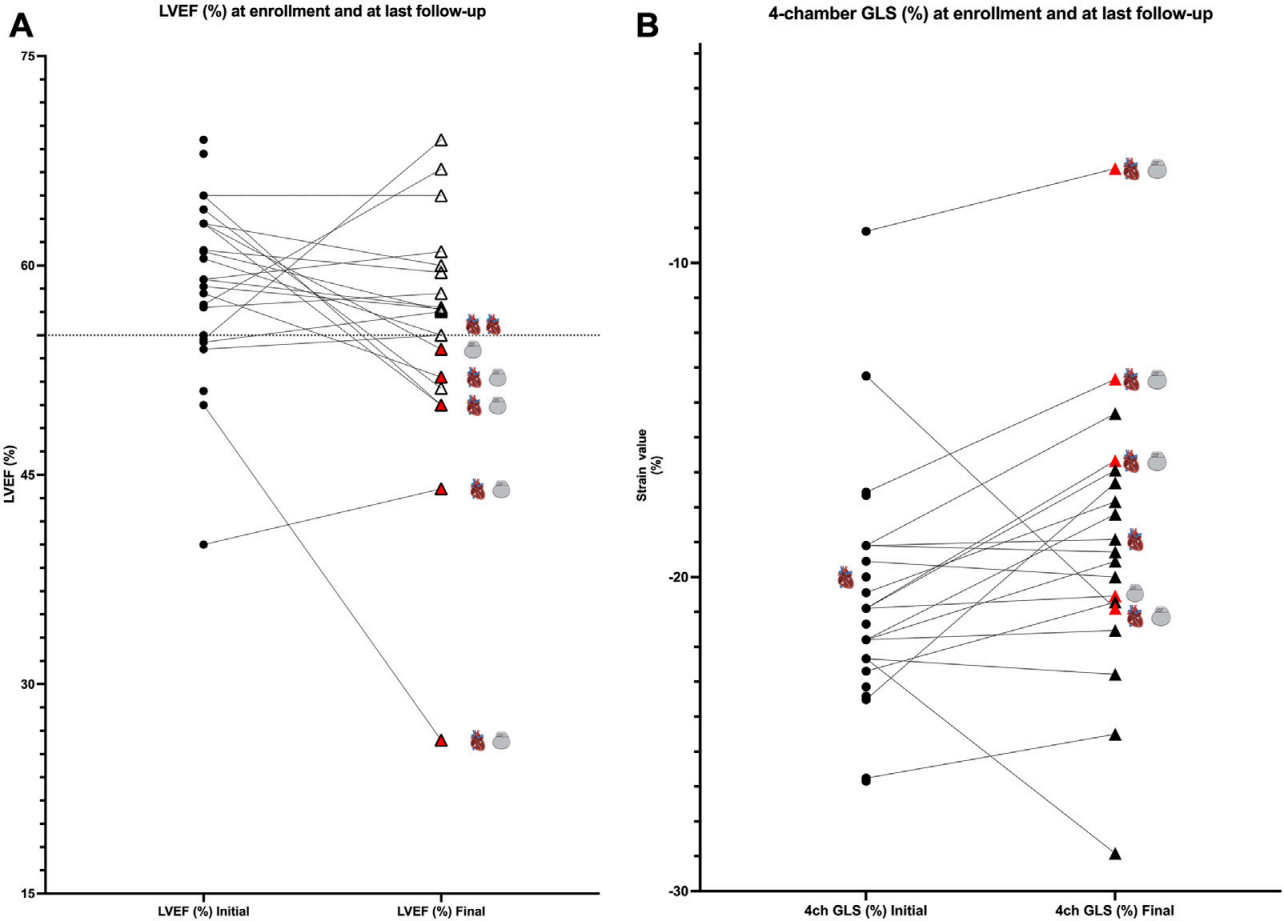
**(A)**: comparison of values at enrollment and at last follow-up; LVEF values (%). Patients are represented as black dots at enrollment, and triangles represent patients at last follow-up. Red triangles show patients requiring an ICD (gray device represented in the graphic). Cardiomyopathy is represented by a heart. Black lines link the same patient in the two different moments of follow-up. **(B)**: comparison of values at enrollment and at last follow-up; four-chamber GLS values (%). Patients are represented as black dots at enrollment and triangles at last follow-up. Red triangles show patients requiring an ICD (gray device represented in the graphic). Cardiomyopathy is represented by a heart. Black lines link the same patient in the two different moments of follow-up.

**FIGURE 3:**
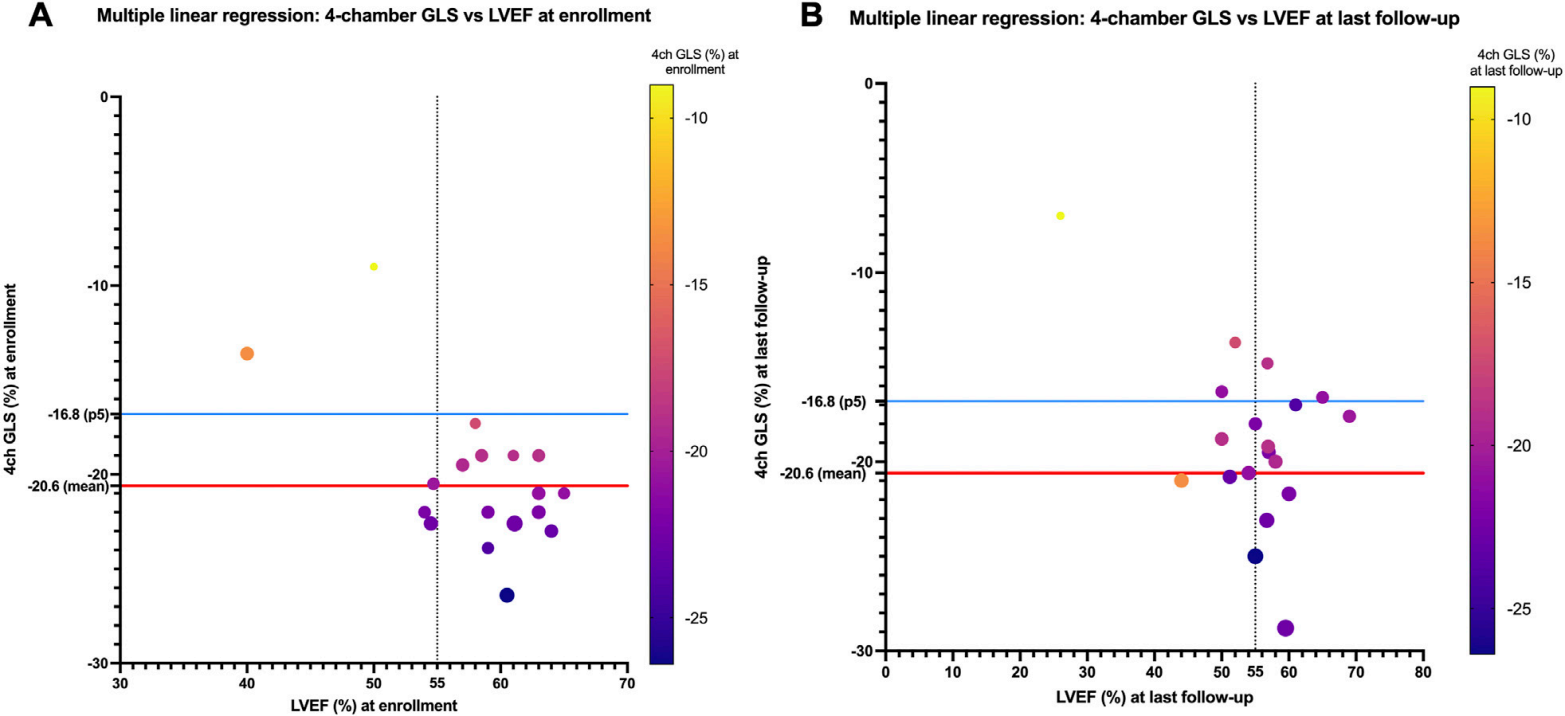
**(A)** Four-chamber GLS values (%) compared with LVEF values (%) for each patient at enrollment. At enrollment, despite normal LVEF (>55%), values below 20.6% are detected (20.6% is the mean normal value published in children). These LVEF values show a decreasing trend at last follow-up **(B)**.

LVEF was compared by the Simpson method in all 28 pediatric patients from enrollment to the end of follow-up, and a global reduction in LVEF during follow-up was found (LVEF 60.75% [IQR of 56–63.5] *versus* 56.75% [IQR of 51.6–59.75], *p* < 0.05; Figure 2A). Patients requiring an ICD (patients 1, 3, 5, 13, and 17) showed worse LVEF values at last follow-up than the rest of the cohort and were older (median age at enrollment of 15 years [IQR of 11–15] and median age at last follow-up of 18 years [IQR of 16–19]) than the rest of the cohort (median age at enrollment of 7 years [IQR of 3–11] and median age at last follow-up of 12 years [IQR of 8–15]). The median time of follow-up was 4 years in the group requiring an ICD (IQR of 4 to 4) and in the rest of the cohort (IQR of 3–5). Five patients showed LVEF values between 45% and 55% (patients 1, 5, 8, 13, and 14). No significant differences in TAPSE were observed when comparing values at enrollment with those at last follow-up (TAPSE of 19.9 mm [IQR of 15.3–22]). The value of TAPSE was ≤17 mm at last follow-up in four of five patients requiring an ICD. GLS (%) analyzed in the apical four-chamber view showed significantly lower values at enrollment than at last follow-up (−21 [IQR of −19 to −22.6] *versus* −17.6 [IQR of −16.3 to −20.6], *p* = 0.01; Figure 2B). At enrollment, GLS values below the mean (<−20.6%) were detected despite normal LVEF values (>55%) in most participants. At last follow-up, these patients showed decreasing GLS and LVEF (Figures 2A, 3B). No other significant findings were detected in the analysis of MAPSE, lateral E/E′ ratio, and septal E/E′ ratio.

Patients that finally developed DCM showed their first neuromuscular symptoms before 2 years of age (early-onset EDMD and L-CMD phenotypes) suggesting an early-onset aggressive form of laminopathy.

#### Arrhythmia and long-term loop recorder monitoring analysis

The ECG analysis is summarized in Table 2. Minor ECG abnormalities were present in 16 patients. Within these minor abnormalities, the following were described: first-degree AVB, wide QRS complex, short PR interval, global QRS low voltage, high QRS voltage V1–V4, QS complex in V1–V2, poor R progression, incomplete right bundle-branch block (IRBBB), abnormal Q waves, sinus bradycardia, and repolarization abnormalities. The most frequent finding (28.5%, eight patients) was global low QRS voltage that presented early; seven of eight cases had neither ventricular dysfunction nor DCM (Supplementary Figures S3A–C). The early-onset group showed the following ECG abnormalities before 2 years of age: global QRS low voltage and poor R progression.

EPS at enrollment showed seven patients (25%) with atrial conduction disorders (short non-sustained atrial tachycardia [*n* = 6] and intermittent atrial standstill [*n* = 1]) that did not merit any further treatment at that moment. No other forms of supraventricular or ventricular tachycardia were induced, and no accessory pathways were detected. HV intervals (time from the proximal His bundle to the ventricular myocardium) were within normal values. When retrospectively analyzing the EPS data in patients who presented with ventricular arrhythmia during follow-up, no abnormalities were observed.

In the ILR monitoring device analysis, malignant arrhythmia (VT/VF) was detected in five cases. (patients 1, 3 [died], 5, 13, and 17). Supplementary Table S3 summarizes the indication and the median age for device implantation. All had normal EPS results at enrollment and were never diagnosed with malignant arrhythmias at our center or from a referring hospital. In all five cases, malignant arrhythmias were detected a few months after enrollment, and they were then considered candidates for ICD implantation (Supplementary Table S3). Two of these patients that were diagnosed with VT through the ILR monitoring device had histories of intermittent pallor and fainting episodes. After ICD implantation, two patients received appropriate shocks. No inappropriate shocks were detected during follow-up. All patients carrying an ICD showed their first neuromuscular symptoms before 2 years of age.

**FIGURE 4:**
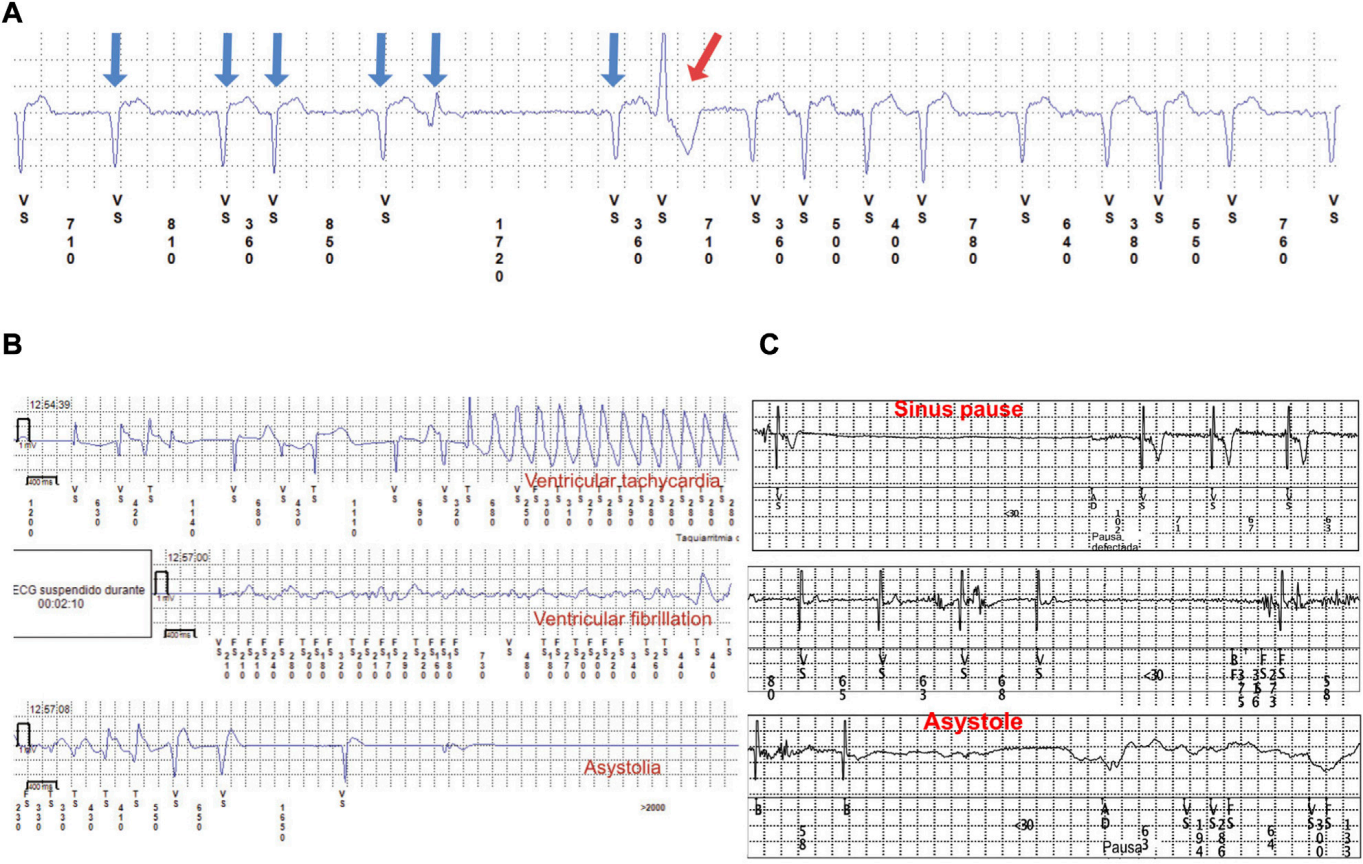
Three ECG traces from the ILR. **(A)** Atrial fibrillation (blue arrows) and a ventricular extrasystole (red arrow). **(B)** Ventricular tachycardia, VF, and asystole from a pediatric patient diagnosed with EDMD. **(C)** Sinus pause and asystole in a 3-year-old patient.

In one participant with an L-CMD phenotype, a premature PM implantation was required because of symptomatic prolonged asystole (patient 2, 2-year-old boy). Patients 1, 3, and 5 (median age of 11 years [IQR of 11–16]) showed intermittent atrial standstill detected in traces from the ILR. During follow-up, atrial fibrillation was diagnosed in four cases (patients 1, 3, 5, and 20; median age at atrial fibrillation diagnosis of 11 years [IQR of 7.5–13.5]), and three were later diagnosed with DCM. Atrial tachycardia was diagnosed in 11 patients (patients 1–7, 9, 13, 20, and 24; median age at atrial tachycardia diagnosis of 11 years [IQR of 6–12]), and 4 were later diagnosed with DCM. Both atrial tachycardia and atrial fibrillation were present simultaneously in four patients. Example electrocardiographic traces from an ILR monitoring device are represented in Figure 4. Neither arrhythmia nor major cardiac events were detected within the patients with mild muscular impairment and late onset after 2 years old of age (patients 8, 15, 16, 18, and 22).

#### Demise

Two patients (2/28, 7.1%) died during follow-up. One (patient 3) was an 18-year-old female with intermittent atrial standstill diagnosed in the EPS and had an ICD due to VT/VF. The patient died because of rapidly progressive heart failure despite optimal treatment. Patient 24 was a 10-year-old female with no cardiovascular involvement who died due to respiratory infection. Both patients were early-onset EDMD, non-ambulant, required NIMV, and showed their first clinical manifestations of muscle weakness before 2 years of age.

### Genetics and genotype-phenotype correlation

All 28 patients carried one rare variant in *LMNA* classified as likely pathogenic or pathogenic following American College of Medical Genetics (ACMG) guidelines and according to currently available data (Supplementary Table S4). Family segregation showed that in 26 cases (92.85%) the rare variant in *LMNA* was *de novo*. Family history of cardiac disease or neurological impairment was present in only two cases (patients 11 [c.1487_1488+9del] and 20 [p.Gln36His]). In both cases, the parents carried the same variant in *LMNA* and had mild neuromuscular impairment and malignant arrhythmias.

Twenty rare *LMNA* variants were identified (18 exonic and 2 intronic). Of all exonic rare variants, 4 were deletions, and 14 were missense. Thirteen rare variants (65%) were classified as likely pathogenic and seven (35%) as definitively pathogenic. Twelve variants were novel (two intronic, two deletions, and eight missense; Supplementary Table S4).

**FIGURE 5:**
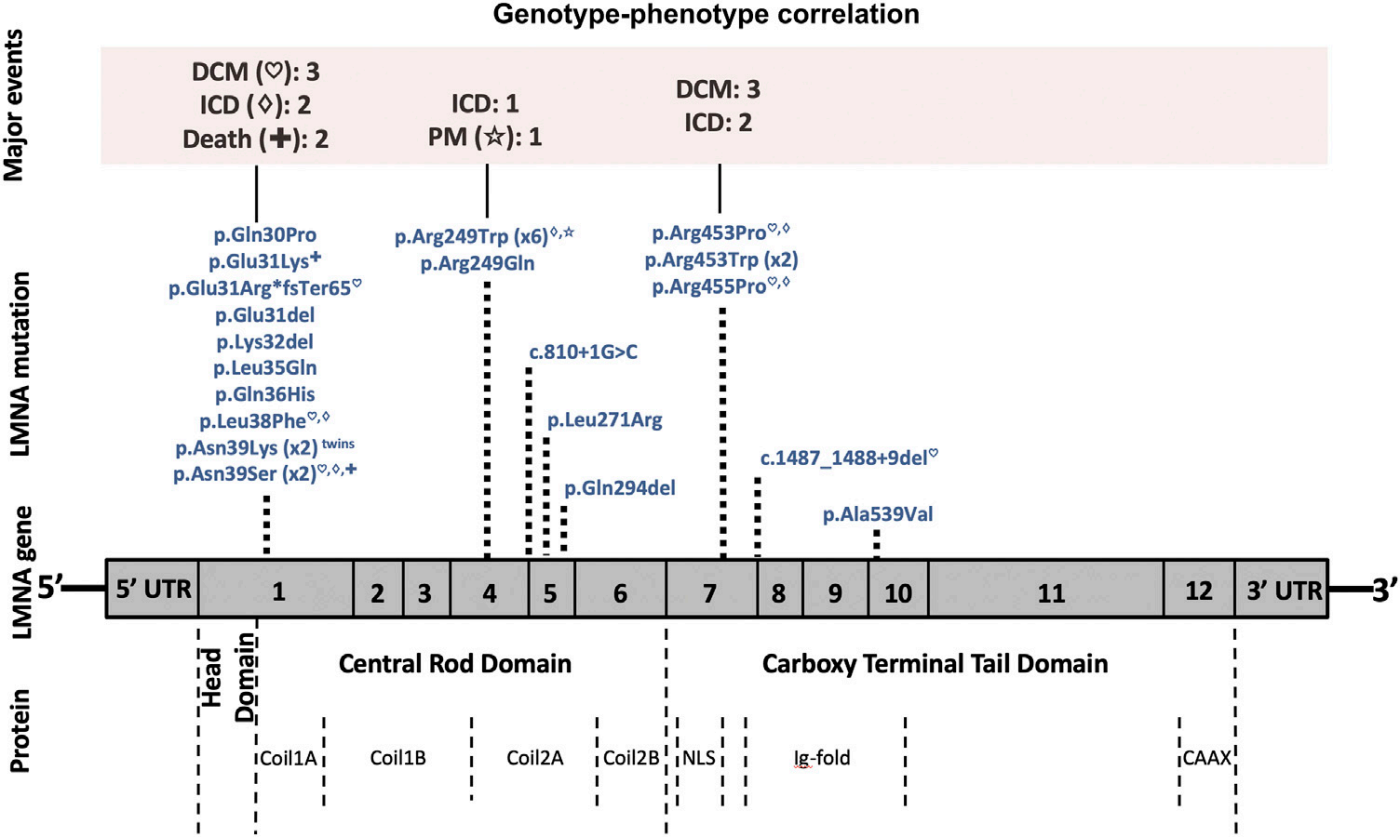
Phenotype–genotype graphic correlation. A schematic representation of the *LMNA* gene is shown. Top, major cardiac events, followed by rare pathogenic/likely pathogenic *LMNA* variants identified in our patients with the corresponding amino acid or nucleotide changes. Bottom, structural protein: the coil 1A–1B and coil 2A–2B constituting the ⍺-helical rod domain. Abbreviations: DCM, dilated cardiomyopathy; ICD, implantable cardioverter defibrillator; PM, pacemaker; NLS, nuclear location signal; Ig, immunoglobulin.

The most prevalent variant identified was p. Arg249Trp (six patients, 21.4%) located in exon 4 of *LMNA*. These six patients were diagnosed with L-CMD (four patients), EDMD (one patient), or lamin-related mild weakness (one patient). One patient showed malignant arrhythmias and required an ICD, and another patient needed a PM due to asystole (both L-CMD with dropped head). No DCM was detected with this rare variant. Figure 5 shows the correlation of genotype to phenotype of all 28 patients. The *LMNA* mutations identified in the six patients with DCM (patient 3 (died), 5, 9, 11, 13 and 17) were p. Asn39Ser, p. Arg453Pro, p. Glu31ArgfsTer65, c.1487_1488+9del, p. Leu38Phe, and p. Arg455Pro. In the five patients with ICDs implanted due to malignant arrhythmias (patients 1, 3 [died], 5, 13, and 17), all carried rare variants often identified in pediatric LMNA-muscular dystrophy patients (p.Arg249Trp, p. Asn39Ser, p. Arg453Pro, p. Leu38Phe and p. Arg455Pro, respectively). Four cases were diagnosed with EDMD and one with L-CMD. The two cases who died (patients 3 [with an ICD] and 24) carried also a rare variant (p.Asn39Ser and p. Glu31Lys, respectively), both located in exon 1 of *LMNA.* Both were diagnosed with early-onset EDMD and showed their first neuromuscular symptoms before 2 years of age.

## Discussion

Laminopathies are a group of ultrarare genetic diseases attributable to pathogenic rare variants in the *LMNA* gene (Worman and Bonne, 2007; Bonne and Quijano-Roy, 2013). Due to its low prevalence, few cases have been diagnosed and reported so far, and there are not enough published data about its natural history. This fact, reinforces the importance of our prospective pediatric international registry including congenital phenotypes. Other multicenter laminopathy studies and expert consensus statement on arrhythmic risk have been published for adult populations (Pasotti et al., 2008; Rijsingen et al., 2012; Haas et al., 2014; Kumar et al., 2016; Peretto et al., 2019; Groh et al., 2022) but did not focus on cardiac involvement in children, as we describe here. Additionally, reports on cardiac impairment in pediatric laminopathies with neuromuscular involvement are lacking, despite that cardiac events can be present at early stages (Benedetti et al., 2007; Maggi et al., 2014; Fan et al., 2020; Groh et al., 2022). Our study offers longer follow-up in a pediatric population and valuable information about cardiac events, neuromuscular phenotypes, and genotype–phenotype correlations.

*LMNA*-related diseases cause cardiac symptoms in children and young adults that gradually worsen to bradyarrhythmias and tachyarrhythmias (Groh, 2012; Rijsingen et al., 2012; Rijsingen et al., 2013; Alexandra et al., 2015; Priori et al., 2015; Groh et al., 2022). Approximately half of patients need a PM or an ICD during adulthood (Taylor et al., 2003; Priori et al., 2015). Cardiac involvement occurs in a high percentage of cases, and implantation of preventative measures, such as an ICD, is recommended in patients with malignant arrhythmias (Pasotti et al., 2008; Rijsingen et al., 2012; Maggi et al., 2014; Groh et al., 2022). Pathogenesis of *LMNA*-related cardiomyopathy remains unclear, but lamin A/C haploinsufficiency may have negative effects on the heart (Wolf et al., 2008). Both fibroblasts from L-CMD patients and myoblasts from L-CMD mouse models demonstrate increased nucleoplasmic localization of lamin A/C compared to controls and EDMD. Mislocalization of nuclear envelope proteins leads to defects in myoblast differentiation, contributing to the more severe phenotype observed in L-CMD. (Bertrand et al., 2020). Clinical recommendations for these patients were identical to those for patients with other cardiomyopathies or heart failure: pharmacologic treatment with neurohormonal antagonists, diuretics, and vasodilators and non-pharmacological treatment with ventricular device therapy, such as early PM or resynchronization therapy for progressive conduction delays and an ICD to prevent SCD (Yancy et al., 2013; Priori et al., 2015; Bozkurt et al., 2016; Yancy et al., 2016; Atalaia et al., 2021). An expert consensus on evaluation and management of arrhythmic risk in neuromuscular disorders, published recently highlighted the especial recommendations for diagnostic testing and risk stratification in adults affected with EDMD or LGMD1B. Within the recommendations in adult population, implantable cardiac monitoring is reasonable even in the setting of a normal 12-lead ECG, ambulatory ECG monitoring and normal echocardiogram. Sustained arrhythmias, AVB and SCD are highly present in these patients and comprehensive risk stratification is needed, including EP study in select patients. Heart transplantation may be considered in certain cases with mild neuromuscular impairment (Taylor et al., 2003; Finsterer et al., 2006; Maggi et al., 2014; Pérez-Serra et al., 2016).

Six pediatric patients carrying a deleterious rare variant in *LMNA* concomitant with congenital heart disease (CHD) were previously found to have no major neuromuscular involvement (Baban et al., 2020) Most cases showed a family history of CHD and/or DCM associated with arrhythmias. Neither CHD nor aortic involvement were found in our cohort. Despite a rare deleterious variant in the *LMNA* gene, this previously reported phenotype is not similar to our study because most of our cases showed severe neuromuscular involvement, and rare *LMNA* variants are *de novo*. The wide range of phenotypes associated with rare pathogenic variants located in the *LMNA* gene are well known. During follow-up in a retrospective series of 15 pediatric patients carrying *LMNA* variants with early-onset neuromuscular symptoms, no major cardiac involvement was described (two cases of supraventricular arrhythmia, no malignant arrhythmias, no DCM, and no sudden death) or specific cardiovascular description (Jędrzejowska et al., 2021). In contrast to theses previous studies, our study characterizes the impact of LMNA variants in pediatric patients with both neuromuscular and cardiovascular involvement.

To our knowledge, no more than 10 reports have focused on pediatric laminopathies with both neuromuscular and cardiac involvement. In a retrospective study, 151 patients carrying a mutation in *LMNA* showed an early-onset phenotype, and the most frequent mutation was p. Arg249Trp; 63% of patients never acquired independent ambulation, and 37% died. Early cardiac interventions (heart medication and/or PM or ICD implantation) were usually associated with earlier respiratory interventions (intermittent positive pressure breathing, non-invasive ventilation of tracheostomy), and clinical severity was positively correlated across the triad of skeletal muscles, respiratory muscles, and myocardium. Correlation to cardiac device placement is less strongly linked with the timing of respiratory interventions and the progression of skeletal muscle weakness. Prospective natural history studies should therefore be conducted to further validate the stratification of L-CMD (Ben Yaou et al., 2021). We performed a prospective cardiac natural history of pediatric patients with *LMNA*-related muscular dystrophy.

Other studies included no more than eight patients, were retrospective, and had no adoption of preventive arrhythmogenic measures or follow-up (Bonne et al., 2000b; Komaki et al., 2011; Pasqualin et al., 2014; Parent et al., 2015; Petillo et al., 2015; Tan et al., 2015; Heller et al., 2017). In our cohort, patients 1, 2, 6, 14, 18, and 28 carried variant p. Arg249Trp, which is present in pediatric patients with arrhythmic complications without major ventricular dysfunction and could be related to neuromuscular severity (Komaki et al., 2011; Ben Yaou et al., 2021). Patient 3 and patient seven carried p. Asn39Ser, which was previously described in two patients (one died of cardiac arrest due to malignant arrhythmias, and one had premature ventricular contractions [PVCs]) (Pasqualin et al., 2014). Patient 8 carried p. Arg249Gln, which was previously reported in children with arrhythmias (Bonne et al., 2000b; Komaki et al., 2011; Heller et al., 2017).

### Cardiac involvement by neuromuscular phenotype

#### Lamin-related congenital muscular dystrophy/dropped head phenotype

Two groups are distinguished depending of the severity of onset, a very early form with arrest of motor development before the age of 6 months (no sitting or walking acquisition) and another with initial normal or subnormal motor milestones and subsequent loss, beginning by a characteristic presentation of loss of head support (dropped head syndrome) (Quijano-roy et al., 2008). All children have a progressive course with an initial rapid decline in cervical and axial tonus strength followed by a period of slower progression or plateau. Respiratory insufficiency is a major complication in the course of both entities, being responsible for early death in the early severe group, as early as the first 2 years of age (if no adequate ventilatory support is provided), or leading to mechanical ventilation which can evolve from non-invasive to invasive ventilation *via* tracheostomy in few years. Cardiac involvement is rarely observed initially in these children and is often subclinical in very young patients, but SCD can occur (Quijano-roy et al., 2008); therefore, routine cardiac follow-up is recommended (Quijano-roy et al., 2008; Lu et al., 2011). Our study revealed only one case (dropped head) with mild DCM with borderline LVEF values diagnosed before 10 years of age. During our follow-up, malignant arrhythmia was detected in one case (patient 1, dropped head) and an ICD was implanted at 12 years of age. Four patients showed atrial tachycardia, and one patient (patient 1, dropped head and non-ambulant) showed simultaneous atrial fibrillation and atrial tachycardia starting at 11 years of age. An ambulant child with dropped head syndrome (patient 2) had asystole and needed a PM. Both patients carried the same pathogenic rare *LMNA* variant p. Arg249Trp, which is the most frequent in pediatric squeletal laminopathies and is associated with worse clinical prognosis (Ben Yaou et al., 2021). These results support the need for close follow-up, which could show subclinical cardiac involvement in pediatric patients diagnosed with L-CMD. In a recent publication, echocardiography abnormalities were identified in 22% of L-CMD patients, and device implantation (ICD and PM) was described in 9% and 7% of the cohort, respectively (Ben Yaou et al., 2021); however, there is a lack information about the cause of death and reason for device implantation (Heller et al., 2017; Ben Yaou et al., 2021).

#### Emery-dreifuss muscular dystrophy

EDMD is the third most prevalent muscular dystrophy, and most patients present with autosomal-dominant EDMD due to *LMNA* (with higher risk of VT and DCM). *EMD* (emerin) gene is less frequent, with X-linked transmission (Bonne et al., 1999; Bonne and Quijano-Roy, 2013). However, more than 60% of EDMD cases have no deleterious variant in *EMD* or *LMNA* genes. Cardiac complications in EDMD patients can be life-threatening and lead to progressive cardiac failure, cardiac conduction disease, and SCD. DCM may occur at an advanced stage, but conduction disease is frequent (complete heart block, silent atria, atrial tachycardia, atrial fibrillation, and VT) (Figures 4B, C). Patients with EDMD are also at risk of cerebral emboli and sudden death (Bonne et al., 1999; Lu et al., 2011; Bonne and Quijano-Roy, 2013; Cattin et al., 2013; Finsterer et al., 2015; Priori et al., 2015; Marchel et al., 2021a; Marchel et al., 2021b; Groh et al., 2022). In our study, early presentation of atrial tachycardia (seven patients), atrial fibrillation (three patients), and different grades of ventricular dysfunction (six patients) that led to heart failure and malignant arrhythmias (four patients) were detected in early-onset EDMD children. Four patients required an ICD because of malignant arrhythmias (between 14 and 18 years old at ICD implantation). Surprisingly, all showed an EPS with no ventricular arrhythmia Inducibility, and the arrythmias were instead detected with the ILR. These malignant arrhythmias corresponded to sustained and non-sustained VT, and all were asymptomatic except patient 3 who exhibited pallor and seizures during VT/VF episodes. As previously described (Steckiewicz et al., 2016; Marchel et al., 2021a), the low rate of symptoms and presentation of atypical symptoms could make the global management of patients with EDMD difficult, worsening the prognosis. Antiaggregant therapy was needed in four patients because of atrial standstill, and one patient (patient 3) presented with an episode of cerebral emboli at 17 years of age, likely of multifactorial origin, before starting treatment. *LMNA* carriers have an increased risk of thromboembolic events, with an inherent risk independent of heart condition (Rijsingen et al., 2012; Rijsingen et al., 2013; Van Rijsingen et al., 2013; Groh et al., 2022) This thromboembolic risk could be related to cardiac dysfunction secondary to DCM and/or cardiac conduction disease, such as silent atria, sinus node dysfunction, sinus pauses, and atrial fibrillation (Groh et al., 2022). Long-term systemic anticoagulation therapy with a vitamin K antagonist and international normalized ratio (INR) target of 2.0–3.0 would be reasonable in DCM patients with arrhythmias, previous thromboembolic events, thrombophilic conditions, or an ejection fraction of ≤25%, but there is no evidence in pediatric laminopathies and traditional algorithms, such as CHA_2_DS_2_-VASc, are inappropriate for children. When silent atria or atrial fibrillation are present in children, long-term anticoagulation therapy is only indicated when there are other risk factors (previous stroke, transient ischemic attack, hypertension, or heart failure). As atrial arrhythmias, immobilization, and heart failure are usually concomitant findings in *LMNA*-related muscular dystrophy patients, long-term systemic anticoagulation therapy may benefit higher-risk patients, for example, those with a LVEF of ≤25% and atrial arrhythmias. If patients have any contraindication to receive vitamin K antagonist or low-molecular-weight heparin, aspirin should be recommended (Monagle, 2012; Giglia et al., 2015). We observed that patients diagnosed with early-onset EDMD are at higher risk of severe cardiac involvement, mainly DCM in those older patients of our cohort, while life-threatening arrhythmias without DCM appear earlier in L-CMD patients. These findings are in agreement with previous studies (Maggi et al., 2014; Fan et al., 2020; Marchel et al., 2021b), but more studies should be performed. Arrhythmic events predict myocardial involvement in pediatric patients carrying a deleterious *LMNA* variant (Bonne et al., 2000b; Komaki et al., 2011; Pasqualin et al., 2014; Parent et al., 2015; Petillo et al., 2015; Tan et al., 2015; Heller et al., 2017; Groh et al., 2022) reinforcing the importance of investigating cardiac involvement in childhood laminopathy cohorts, even in the absence of clear neuromuscular involvement (Baban et al., 2020). Our results showed a high rate of arrhythmias at pediatric age, so early detection and treatment for each case according to guidelines is critical to preventing complications, improving prognosis, and avoiding death.

#### Limb-girdle muscular dystrophy type 1B

LGMD1B is a subtype of limb–girdle muscular dystrophy that presents with progressive shoulder and hip gridle weakness with prior effects on the inferior limbs *versus* the upper limbs. LGMD1B is autosomal dominant and associated with AV conduction defects, supraventricular arrhtyhmias, and ventricular arrthythmias, but late-onset DCM (Muchir et al., 2000; Lu et al., 2011) and SCD (Finsterer et al., 2015; Groh et al., 2022) have been reported. Evaluation and management of arrhythmic risk are similar to EDMD patients as published in expert consensus statement in adult population with neuromuscular disorders (Groh et al., 2022). We saw no patients of pediatric age (8 and 16 years old at last control) diagnosed with DCM and no ventricular dysfunction, and the ILR registries showed no arrhythmias.

#### LMNA-related atypical phenotype with mild weakness

Patients with *LMNA*-related atypical or ‘undefined’ phenotype were identified in this study because they did not reach phenotypic criteria for L-CMD, EDMD, and LGMD1B phenotypes. They showed an intermediate phenotype between ‘dropped head’ and ‘early EDMD’ (mild weakness, selective hypotrophy and weakness in quadriceps and elbow flexors, mild retractions in elbows, hamstrings, or paraspinal muscles, and mild weakness in neck flexors and foot extensors) as it has been showed in the literature, confirming that these phenotypes are not different entities but probably a continuum (Maggi et al., 2016). Some patients have shown DCM in the literature (Maggi et al., 2014). Our study included two patients (10 and 18 years old at enrollment, 5 and 3 years of follow-up respectively) that showed late-onset muscular manifestations, and none presented with cardiovascular involvement during follow-up at pediatric age. These findings support the idea that early-onset, severe muscular dystrophy is associated with earlier and more severe cardiac involvement.

#### Early-onset clinical manifestations before 2 years of age

Patients with early-onset neuromuscular impairment could show early cardiac manifestations, as published recently in a retrospective review (Ben Yaou et al., 2021). Twenty-three of the 28 patients included in our cohort showed early-onset muscle impairment before 2 years of age. Those patients diagnosed with DCM during follow-up had an early-onset phenotype (L-CMD and early-onset EDMD), and no patients with late-onset phenotypes were candidates for an ICD during follow-up. These findings suggest that earlier onset neuromuscular impairment may lead to a worse cardiac phenotype and higher risk of SCD. It would therefore be reasonable to implant an ILR device in LMNA children presenting inf the first 2 years of life with muscle weakness.

#### Echocardiographic analysis during follow-up

Despite a preserved LVEF, longitudinal myocardial strain analysis speckle tracking could show different patterns indicative of early abnormal segmental strain deformation (especially septal strain compared with non-septal strain), post-systolic deformation, and mechanical dispersion (Hasselberg et al., 2014; Haugaa et al., 2015; Boer et al., 2017). (Hasselberg et al., 2014; Haugaa et al., 2015; Boer et al., 2017). This myocardial strain analysis is well reported in other neuromuscular diseases, such as cardiomyopathy-related Duchenne muscular dystrophy. In our study, echocardiographic analysis during follow-up suggested that pediatric laminopathies with neuromuscular involvement are linked to relatively rapid progression to DCM and low LVEF values in a few years. Despite well-preserved LVEF values, four-chamber GLS values at an early age could identify patients that may need an ICD at pediatric age. To our knowledge, this is the first publication that suggests this relationship early during childhood (Supplementary Figure S4). The RV may be involved in *LMNA*-related cardiomyopathy with or without neuromuscular disease (Quarta et al., 2012; Forleo et al., 2015; Peretto et al., 2019; Marchel et al., 2021b). The lower TAPSE values found in patients that eventually needed an ICD could be associated with RV involvement described previously in adult series with an EDMD phenotype, (Buckley et al., 1999; Marchel et al., 2021a; Marchel et al., 2021b), but the suboptimal acoustic window did not allow for an exhaustive analysis to describe a strong trend or relationship.

#### Arrhythmia and death

Typical early manifestations of arrhythmia in an ECG are flat P wave, AVB, and supraventricular and ventricular arrhythmias (Maggi et al., 2016; Finocchiaro et al., 2020). Other published ECG features include LV hypertrophy data, ST depression, wide QRS complex, and P terminal force. Septal remodeling data in leads V1–V3 seem to be frequent (Ollila et al., 2017), such as Q waves in V1–V2, fragmented QRS in V2–V3, RV1>RV2, RV2>RV3, and poor R wave progression. During follow-up, 12-lead ECG and 24-h Holter monitoring are recommended at least yearly. In our study, first degree AVB, flat P wave, LV hypertrophy data, fragmented and wide QRS complex, poor R wave progression, global low QRS voltage (Supplementary Figure S3C), IRBBB, RBBB, and LBBB were detected during follow-up, in agreement with previous reports (Maggi et al., 2016; Ollila et al., 2017; Finocchiaro et al., 2020). According to our data, ILR with home monitoring is useful for early detection of potential life-threatening arrhythmias, which are usually asymptomatic (Heller et al., 2017).

*LMNA* carriers may experience DCM and SCD before they experience overt heart failure, as ∼30% of patients will have SCD and another 30% will develop congestive heart failure. Males carrying a deleterious *LMNA* variant have worse prognosis because of malignant arrhythmias and heart failure. Laminopathies are the third neuromuscular disease in which SCD is frequently reported (Finsterer and St, 2016). In our pediatric study, and in agreement with previously published reports in adults, malignant arrhythmias are related in four/five cases to male sex, but death was related to female sex in early-onset EDMD (Marchel et al., 2021b). Our study may not be large enough to make conclusions about these trends. Atrial conduction disease and malignant arrhythmias are detected during pediatric follow-up that may lead to thromboembolism, heart failure, and SCD, especially in pediatric patients diagnosed with early-onset EDMD and L-CMD. Because there are no detailed recommendations on cardiovascular management in consensus guidelines (Wang et al., 2010), a specific protocol to detect arrhythmias is needed in this pediatric population because SCD may occur before heart failure (Berlo et al., 2005; Lu et al., 2011). We propose a tentative protocol (Supplementary Protocol S1).

The role of EPS in pediatric neuromuscular disease has not been reported; however, it would be reasonable to include it during follow-up childhood and adulthood if any symptoms (typical or atypical) occur because it could be related to potentially malignant arrhythmia. As suggested in adult population with EDMD or LGMD1B, when individuals exhibits symptoms consistent with bradycardia or VT-related symptoms or ECG shows conduction disorder, EP study may be considered for risk stratification for sustained arrhythmias, AVB and SCD (Groh et al., 2022). Our cohort includes early-onset phenotypes in the majority of patients. In our prospective cohort, early-onset patients characterized by their first neuromuscular impairment before 2 years of age seem to have a direct relationship with major and minor cardiac events, regardless of the final neuromuscular phenotype, as previously suggested (Ben Yaou et al., 2021). We therefore propose a clinical protocol for a comprehensive cardiac assessment of pediatric patients with *LMNA*-related muscular dystrophy.

#### Limitations

Our study has some limitations. Our hospital is an international reference center for neuromuscular diseases, and the most severe patients are referred to our institution. We had missing echocardiographic data because some patients were recruited internationally and there was no follow-up. *LMNA*-related muscular dystrophy is a very rare, often underdiagnosed, disease; therefore, all collected data are of great value.

Additionally, cardiac magnetic resonance imaging (CMRI) was not consistently available for all patients during follow-up. CMRI with late gadolinium enhancement imaging is recommended to study the presence and distribution of myocardial fibrosis. Fibrosis is frequently located along the interventricular septum, according to the worst values in the myocardial strain analysis by speckle tracking. Underlying septal fibrosis could explain the high incidence of ventricular arrhythmias, cardiac conduction delays, and ventricular dysfunction (Holmström et al., 2011; Muscogiuri, 2017). Biomarkers may help during cardiac follow-up, such as N-terminal probrain natriuretic peptide (NT proBNP), a well-recognized biomarker that increases when ventricular function worsens (Yancy et al., 2016; Marchel et al., 2021b). In our series, NT proBNP was not homogeneously analyzed, and it might help in some cases with a high risk of ventricular dysfunction. As published previously, proteomic analysis of plasma samples could be used in the future to identify individuals with a high risk of sudden death with *LMNA*-related cardiomyopathy (Izquierdo et al., 2016). Our study also lacks analysis of other genes that may be implicated in laminopathies or related muscular diseases. In the future, whole-exome sequencing and/or whole-genome sequencing can be used to identify new alterations in any region of genome.

## Conclusion

Malignant arrhythmias and SCD in *LMNA*-related muscular dystrophy occurs frequently, but no comprehensive studies focused on early identification, adoption of preventative measures, and follow-up have been performed. ILR with home monitoring identified five cases (17%) with malignant arrhythmias, and ICDs were implanted for prevention. Two cases with an ICD showed appropriate shocks. ILR may be critical for early diagnosis of life-threatening arrhythmias in laminopathies with an early-onset neuromuscular phenotype. Remote home monitoring helps for close follow-up. Echocardiographic follow-up, including myocardial strain analysis, might be helpful to identify worse prognosis in patients because DCM is present early before adulthood. Specific clinical guidelines that include management in children and emphasize the use of ILR are needed to standardize treatment and mitigate the risk of SCD, especially in those with early-onset phenotypes.

## Data Availability

The original contributions presented in the study are included in the article/Supplementary Material, further inquiries can be directed to the corresponding author.

## Abbreviations

AV, atrioventricular; AVB, atrioventricular block; CHD, congenital heart disease; CMRI, cardiac magnetic resonance imaging; DCM, dilated cardiomyopathy; EDMD, Emery–Dreifuss muscular dystrophy; ECG, electrocardiogram; EPS, electrophysiological study; GLS, global longitudinal strain; HCM, hypertrophic cardiomyopathy; ICD, implantable cardioverter defibrillator; ILR, implantable loop recorder; IQR, interquartile range; IRBBB, incomplete right bundle-branch block; LBBB, left bundle-branch block; LGMD1B, limb–girdle muscular dystrophy 1B; L-CMD, *LMNA*-related congenital muscular dystrophy; LV, left ventricle; LVEF, left ventricular ejection fraction; MAPSE, mitral annular plane systolic excursion; NIMV, non-invasive mechanical ventilation; NT proBNP, N-terminal probrain natriuretic peptide; PVC, premature ventricular contractions; RBBB, right bundle-branch block; RV, right ventricle; SCD, sudden cardiac death; TAPSE, tricuspid annular plane systolic excursion; VT, ventricular tachycardia; VF, ventricular fibrillation.
