# Supplementary figures and images for: Characterization of cardiac involvement in children with LMNA-related muscular dystrophy
Source: Front Cell Dev Biol. 2023 Mar 10;11:1142937. doi: 10.3389/fcell.2023.1142937 (PMC10036759; doi:10.3389/fcell.2023.1142937)

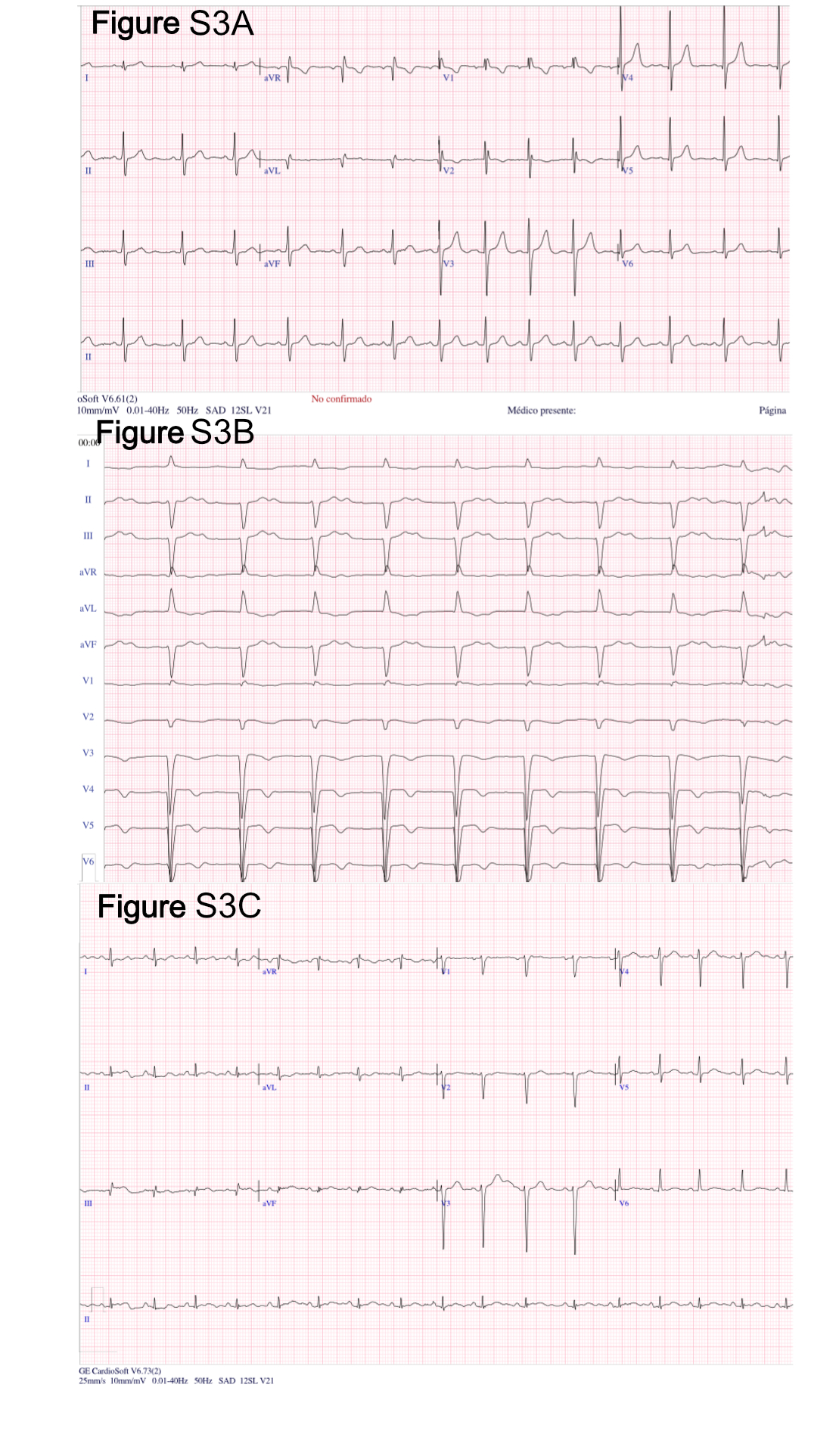

Supplement: Supplementary file 1 [file Image3.JPEG]

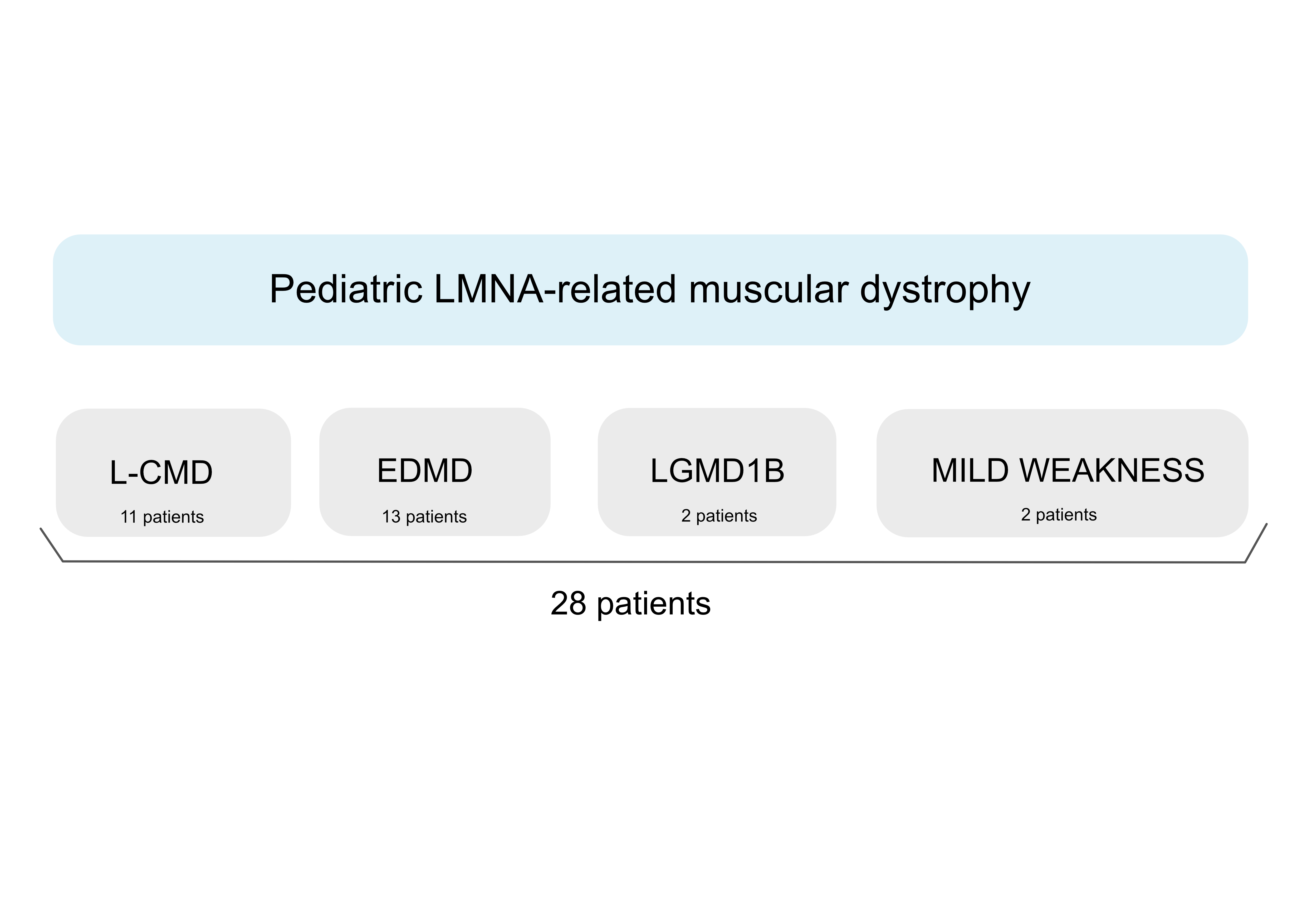

Supplement: Supplementary file 3 [file Image1.JPEG]

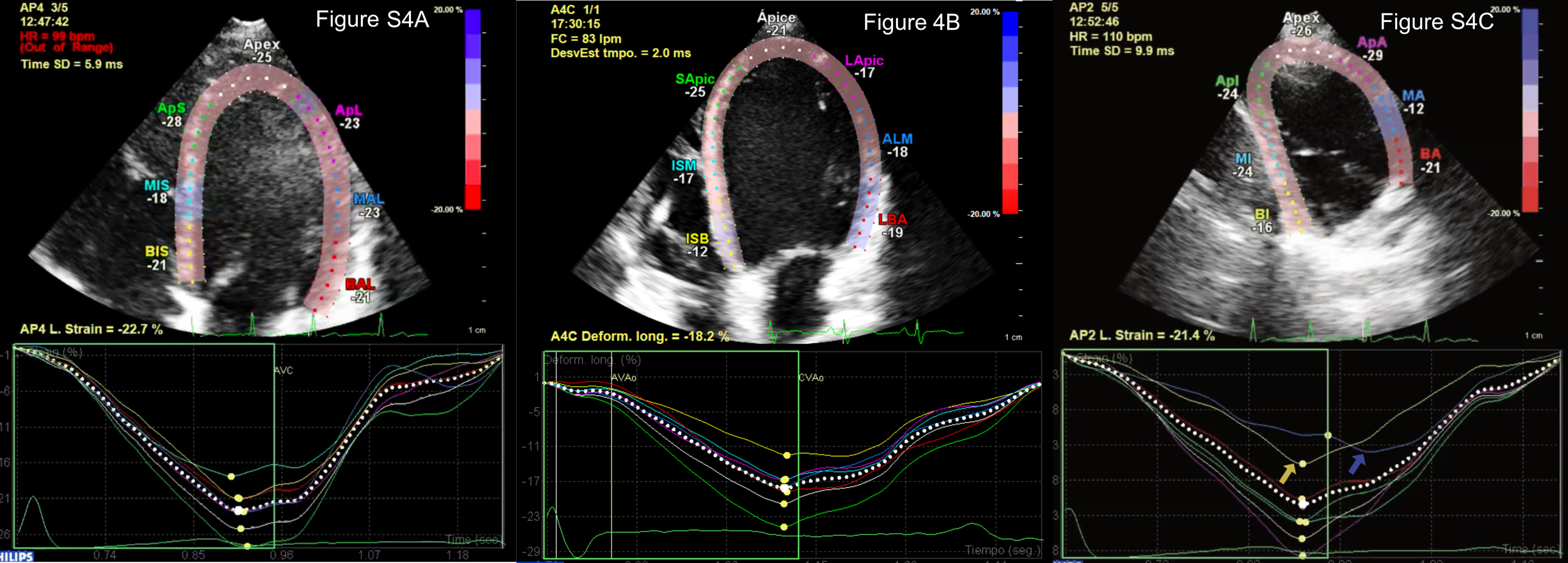

Supplement: Supplementary file 4 [file Image4.JPEG]

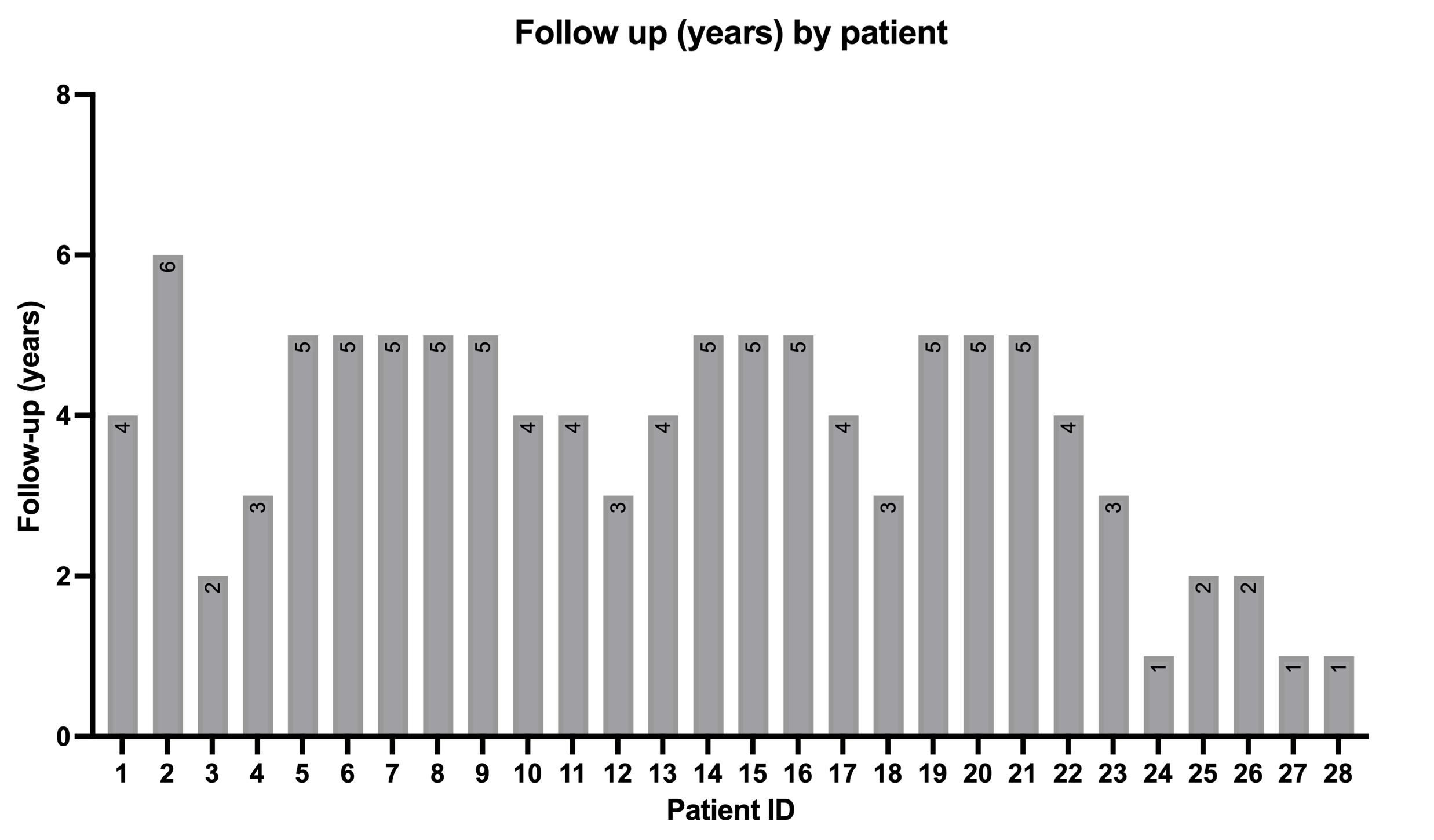

Supplement: Supplementary file 5 [file Image2.JPEG]
