# Supplementary material for: Characterization of cardiac involvement in children with LMNA-related muscular dystrophy
Source: Front Cell Dev Biol. 2023 Mar 10;11:1142937. doi: 10.3389/fcell.2023.1142937 (PMC10036759; doi:10.3389/fcell.2023.1142937)
Supplement: Supplementary file 2 [file Table1.docx]

| **Table S1.** Cohorts in previous published series | | | | |
| --- | --- | --- | --- | --- |
| ***LMNA*-related muscular disease** | **Italy** | | **Chinese** | **Our**  **cohort** |
|  | **Benedetti, 2004** | **Maggi, 2017** | **Fan, 2019** |  |
| **EDMD** | 55.55% | 21% | 38.1% | 46.43% |
| **L-CMD** | 14.82% | 33% | 48.8% | 39.29% |
| **LGMD1B** | 29.63% | 46% | 13.1% | 7.14% |
| **Mild weakness** | . | . | . | 7.14% |
| Comparison between the percentage of *LMNA*-related muscular disease published in other series and the percentage found in our series. Abbreviations: EDMD, Emery–Dreifuss muscular dystrophy; L-CMD, *LMNA*-related congenital muscular dystrophy; LGMD1B, Limb–girdle muscular dystrophy type 1B. | | | | |
