# Supplementary material for: Characterization of cardiac involvement in children with LMNA-related muscular dystrophy
Source: Front Cell Dev Biol. 2023 Mar 10;11:1142937. doi: 10.3389/fcell.2023.1142937 (PMC10036759; doi:10.3389/fcell.2023.1142937)
Supplement: Supplementary file 6 [file Table2.docx]

| **Table S2.** Echocardiographic findings in the cohort | | | | |
| --- | --- | --- | --- | --- |
| **Variable** | | **At**  **Enrollment**  **Median [IQR]** | **Final**  **follow-up**  **Median [IQR]** | ***p*** |
| ***LV systolic function*** | LVEF  (%) | 60.75  [56-63.5]  n=28 | 56.75  [51.6-59.75]  n=20 | **0.04** |
|  | GLS  (%) | -21%  [-19 ;-22.6]  n=28 | -17.6%  [-16.3;-20.6]  n=13 | **0.01** |
|  | MAPSE  (mm) | 11.75  [9.5-12.95]  n=28 | 11  [9.1-12]  n=20 | 0.24 |
| ***LV diastolic function*** | Lateral E/E’  ratio | 5.8  [5.01-7.22]  n= 28 | 5.9  [5.3-7.9]  n=13 | 0.40 |
|  | Septal E/E’  ratio | 7.73  [6.37-8.9]  n= 28 | 7.3  [6.42-9.3]  n=13 | 0.88 |
| ***RV systolic function*** | TAPSE  (mm) | 19.9  [15.3-22]  n=27 | 18  [14-19.5]  n=13 | 0.09 |
| Statistical analysis of the echocardiographic data. The data in the files show echocardiographic analysis: LV systolic function, LV diastolic function, and RV systolic function. The data in the columns show the compared groups in two times: all patients at enrollment versus final follow-up; non-ICD group versus ICD group at enrollment and at final follow-up; non-DCM group versus DCM group at enrollment and at final follow-up. The *P* value for each group is shown in a separate column. Abbreviations: LVEF, left ventricular ejection fraction; LV, left ventricle; RV, right ventricle; GLS, global longitudinal strain; TAPSE, tricuspid annular plane systolic excursion; ICD, implantable cardiac defibrillator; DCM, dilated cardiomyopathy. | | | | |
