# Supplementary material for: Characterization of cardiac involvement in children with LMNA-related muscular dystrophy
Source: Front Cell Dev Biol. 2023 Mar 10;11:1142937. doi: 10.3389/fcell.2023.1142937 (PMC10036759; doi:10.3389/fcell.2023.1142937)
Supplement: Supplementary file 7 [file Table3.docx]

| **Table S3. Age at device implantation** | | | |
| --- | --- | --- | --- |
| Patient’s number | Device | Indication for device | Age at device implantation *(years)*  *Median age: 14.5 (12-17)* |
| 1 | ICD | NSVT | 12 |
| 2 | PM | Asystole | 2 |
| 3 | ICD | NSVT | 17 |
| 5 | ICD | NSVT | 14 |
| 13 | ICD | NSVT | 18 |
| 17 | ICD | NSVT | 15 |
| Reason for indication for a device and class of device in the cohort of study. Median age was 14.5 years (IQR of 12 to 17 years). Patient number is indicated in the first column. The other columns summarize the type of device, the indication for the device, and the age at device implantation. The younger patient was 2 years old and needed a PM because of an asystole detected in the ILR monitoring device. Abbreviations: ICD, implantable cardiac defibrillator; PM, pacemaker; NSVT, non-sustained ventricular tachycardia. | | | |
