# Supplementary material for: Characterization of cardiac involvement in children with LMNA-related muscular dystrophy
Source: Front Cell Dev Biol. 2023 Mar 10;11:1142937. doi: 10.3389/fcell.2023.1142937 (PMC10036759; doi:10.3389/fcell.2023.1142937)
Supplement: Supplementary file 8 [file Table4.docx]

| **Table S4. LMNA genetic data and classification** | | | | | | | |
| --- | --- | --- | --- | --- | --- | --- | --- |
| **Patient** | **Nucleotide Change** | **Protein Change** | **dbSNP** | **gnomAD**  **(MAF%)** | **ClinVar**  **(Disease)** | **HGMD**  **(Disease)** | **ACMG**  **Score** |
| 1 | c.745C>T | p.Arg249Trp | rs121912496 | NA | LP  Lamin-Related Congenital Muscular Dystrophy | CM083718  DM,  Muscular Dystrophy | LP |
| 2 | c.745C>T | p.Arg249Trp | rs121912496 | NA | LP  Lamin-Related Congenital Muscular Dystrophy | CM083718  DM,  Muscular Dystrophy | LP |
| 3 | c.116A>G | p.Asn39Ser | rs57983345 | NA | P  Charcot-Marie-Tooth, Type 2 | CM083713  DM,  Muscular Dystrophy | P |
| 4 | c.91-93delGAG | p.Glu31del | rs864309525 | NA | P  Charcot-Marie-Tooth, Type 2 | CD156162  DM,  Muscular Dystrophy | LP |
| 5 | c.1358G>C | p.Arg453Pro | rs267607598 | NA | NA | CM083716  DM,  Muscular Dystrophy | LP |
| 6 | c.745C>T | p.Arg249Trp | rs121912496 | NA | LP  Lamin-Related Congenital Muscular Dystrophy | CM083718  DM,  Muscular Dystrophy | LP |
| 7 | c.116A>G | p.Asn39Ser | rs57983345 | NA | P  Charcot-Marie-Tooth, Type 2 | CM083713  DM,  Muscular Dystrophy | P |
| 8 | c.746G>A | p.Arg249Gln | rs59332535 | NA | P  Charcot-Marie-Tooth, Type 2 | CM1617006  DM,  Emery-Dreifuss | P |
| 9 | c.91delG | p.Glu31ArgfsTer65 | NA | NA | NA | NA | P |
| 10 | c.89A>C | p.Gln30Pro | NA | NA | NA | NA | LP |
| 11 | c.1487_1488+9del | . | NA | NA | NA | CD1711480  DM,  Muscular Dystrophy | P |
| 12 | c.1616C>T | p.Ala539Val | NA | NA | NA | NA | LP |
| 13 | c.112C>T | p.Leu38Phe | NA | NA | NA | NA | LP |
| 14 | c.745C>T | p.Arg249Trp | rs121912496 | NA | LP  Lamin-Related Congenital Muscular Dystrophy | CM083718  DM,  Muscular Dystrophy | LP |
| 15 | c.812T>G | p.Leu271Arg | NA | NA | NA | NA | LP |
| 16 | c.880_882delCAG | p.Gln294del | NA | NA | NA | NA | LP |
| 17 | c.1364G>C | p.Arg455Pro | rs267607597 | NA | NA | CM111722  DM,  Muscular Dystrophy | LP |
| 18 | c.745C>T | p.Arg249Trp | rs121912496 | NA | LP  Lamin-Related Congenital Muscular Dystrophy | CM083718  DM,  Muscular Dystrophy | LP |
| 19 | c.810+1G>C | . | rs267607632 | NA | P | NA | P |
| 20 | c.108G>T | p.Gln36His | NA | NA | NA | NA | LP |
| 21 | c.104T>A | p.Leu35Gln | NA | NA | NA | NA | LP |
| 22 | c.1357C>T | p.Arg453Trp | rs58932704 | NA | P  Charcot-Marie-Tooth, Type 2 | CM990813  DM,  Muscular Dystrophy | LP |
| 23 | c.1357C>T | p.Arg453Trp | rs58932704 | NA | P  Charcot-Marie-Tooth, Type 2 | CM990813  DM,  Muscular Dystrophy | LP |
| 24 | c.91G>A | p.Glu31Lys | rs1228406418 | NA | P  Charcot-Marie-Tooth, Type 2 | CM123360  DM,  Muscular Dystrophy | LP |
| 25,26^twins^ | c.117T>G | p.Asn39Lys | NA | NA | NA | CM156123  DM,  Muscular Dystrophy | LP |
| 27 | c.94_96delAAG | p.Lys32del | rs60872029 | NA | P  Charcot-Marie-Tooth, Type 2 | CD033712  DM,  Muscular Dystrophy,  Emery-Dreifuss | P |
| 28 | c.745C>T | p.Arg249Trp | rs121912496 | NA | LP  Lamin-Related Congenital Muscular Dystrophy | CM083718  DM,  Muscular Dystrophy | LP |
| List of *LMNA* variants and related features found in our pediatric cohort. The nucleotide and protein changes, predictions, categorization in the HGMD database, and the score otorged classified as pathogenic or likely pathogenic are presented for each case. Abbreviations: dbSNP, single nucleotide polymorphism database; GnomAD (MAF%), genome aggregation database (minor allele frequency %); ClinVar: clinically relevant variation database; HGMD, the Human Gene Mutation Database; ACMG score, the American College of Medical Genetics and Genomics score; NA, not available. | | | | | | | |
