# Supplementary material for: Characterization of cardiac involvement in children with LMNA-related muscular dystrophy
Source: Front Cell Dev Biol. 2023 Mar 10;11:1142937. doi: 10.3389/fcell.2023.1142937 (PMC10036759; doi:10.3389/fcell.2023.1142937)
Supplement: Supplementary file 9 [file DataSheet1.docx]

**Protocol S1:**

**Suggested protocol on cardiovascular management in children with *LMNA*-related muscular dystrophy**

There is a lack of information on monitoring cardiac involvement in presumably healthy children with *LMNA*-related muscular dystrophy. From our results, it is reasonable to propose the following recommendations for management to detect early cardiac dysfunction and arrhythmias:

- Basal 12-lead ECG: at diagnosis and every 6 months.
- Twenty-four-hour Holter monitoring: at diagnosis and every year.
- ILR monitoring device: due to high incidence of arrhythmias (most asymptomatic) and published studies of SCD, the implantation of a long-term loop recorder should be considered to detect arrhythmias early and prevent malignant arrhythmias, treat them, and prevent sudden death, especially in patients with early-onset skeletal muscular weakness.
- Echocardiography, including strain analysis, at diagnosis and yearly. If arrhythmia or depressed ejection fraction are detected, echocardiography is recommended every 6 months or earlier if needed.
- EPS: perform an EPS in those with suspected arrhythmias that could benefit from cardiac ablation and study those patients at risk of malignant arrhythmias and atrial arrhythmias meanable to provoking cerebral emboli. Brachial venous access should be considered for patients with difficult femoral access.
- An implantable defibrillator should be considered if VT is detected and especially before severe ventricular dysfunction appears. A cardiac resynchronization device should also be considered early.
- CMRI with late gadolinium enhancement imaging is recommended to study the presence and distribution of atrial and myocardial fibrosis that could explain the high incidence of ventricular arrhythmias, cardiac conduction delays, and ventricular dysfunction.
- Consider using NT-proBNP as a cardiac biomarker yearly and when cardiac dysfunction is suspected.
- Nonurgent surgical procedures, such as in other neuromuscular diseases with potential cardiac involvement, and deep cardiac evaluation should be performed before any programmed procedure.
